# Thermodynamic origins of two-component multiphase condensates of proteins†

**DOI:** 10.1039/d2sc05873a

**Published:** 2023-01-25

**Authors:** Pin Yu Chew, Jerelle A. Joseph, Rosana Collepardo-Guevara, Aleks Reinhardt

**Affiliations:** a Yusuf Hamied Department of Chemistry, University of Cambridge Cambridge CB2 1EW UK rc597@cam.ac.uk ar732@cam.ac.uk; b Department of Physics, University of Cambridge Cambridge CB3 0HE UK; c Department of Genetics, University of Cambridge Cambridge CB2 3EH UK

## Abstract

Intracellular condensates are highly multi-component systems in which complex phase behaviour can ensue, including the formation of architectures comprising multiple immiscible condensed phases. Relying solely on physical intuition to manipulate such condensates is difficult because of the complexity of their composition, and systematically learning the underlying rules experimentally would be extremely costly. We address this challenge by developing a computational approach to design pairs of protein sequences that result in well-separated multilayered condensates and elucidate the molecular origins of these compartments. Our method couples a genetic algorithm to a residue-resolution coarse-grained protein model. We demonstrate that we can design protein partners to form multiphase condensates containing naturally occurring proteins, such as the low-complexity domain of hnRNPA1 and its mutants, and show how homo- and heterotypic interactions must differ between proteins to result in multiphasicity. We also show that in some cases the specific pattern of amino-acid residues plays an important role. Our findings have wide-ranging implications for understanding and controlling the organisation, functions and material properties of biomolecular condensates.

## Abstract 2

We develop a computational method integrating a genetic algorithm with a residue-level coarse-grained model of intrinsically disordered proteins in order to uncover the molecular origins of multiphase condensates and enable their controlled design.

## Graphical abstract

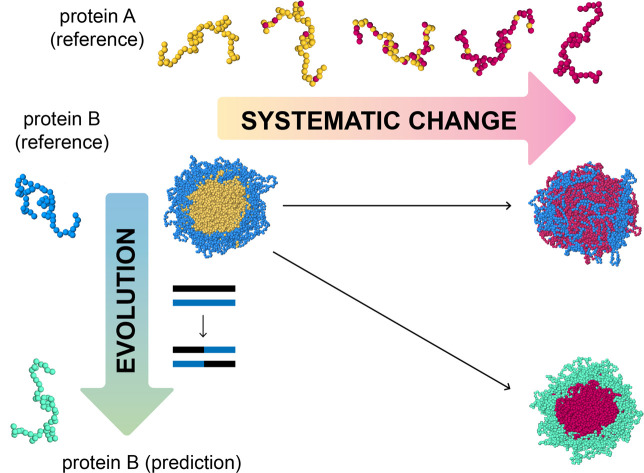

## 1 Introduction

Biomolecular condensates are involved in controlling many aspects of cell biology and pathology. By dynamically segregating particular biomolecules,^1,2^ condensates help create intracellular micro-environments that contribute to the regulation of chemical reactions^3,4^ and mediate a variety of fundamental biological processes, from cell signalling^5–9^ to RNA metabolism,^10–12^ response to stress,^13–17^ regulation of transcription^18–23^ and DNA repair.^24–26^ Moreover, dysregulation of biomolecular phase separation has been associated with a growing list of diseases from cancer to neurodegeneration.^27–32^ The link between aberrant liquid–liquid phase separation (LLPS) and disease highlights the desirability of developing new tools to manipulate the properties of condensates to bypass pathologies. Anti-cancer drugs have already been shown to concentrate preferentially into condensates,^33^ and some small molecules can modulate LLPS,^34^ making biomolecular condensates potential drug targets.

A number of biomolecular condensates *in vitro* and in cells have been observed to display multiphase architectures: structural heterogeneity over mesoscopic length scales, where immiscible phases with different compositions coexist within the same liquid droplet. *In vitro*, multiphase droplets have been observed in various multi-component mixtures, all involving RNA and at least two different proteins, *e.g.* those of poly(PR) and RNA homopolymers,^35^ of polyR, polyK and polyU,^36^ and of prion-like and arginine-rich polypeptides, and RNA.^37^ Inside cells, a prime example is the nucleolus, a highly multi-component system, which exhibits a multilayered architecture^38^ that is thought to be important for sequential processing of nascent rRNA transcripts.^10^ Similar internal structuring is also found in stress^13^ and P granules.^39–41^ The presence of multiple condensed phases in a single condensate may reflect different biological processes taking place in physically separated regions within the same compartment.^42,43^

The emergence of a multiphase organisation has been associated with the physicochemical diversity of the various molecular components. One hypothesis is that multiphase condensates emerge in multicomponent systems when there is competition for a shared binding partner.^36,37^ For instance, competing protein–protein and protein–RNA interactions can provide a regulatory mechanism for the organisation of multiphase condensates.^37^ Simulations of multi-component systems^44,45^ have further revealed that the phase boundary for demixed phases is sensitive to the variance of intermolecular interaction strengths: if it is sufficiently large, multiple distinct phases can form.^45^ From the physicochemical point of view, the formation of an interface between two phases is thermodynamically unfavourable and must be compensated for by free-energetically favourable interactions in the demixed system. The extent to which an interface is unfavourable is quantified by the interfacial free-energy density, which gives the free-energy penalty per unit area of the interface. Unsurprisingly, the various forms of structuring and morphological patterns of multiphase condensates have been suggested to be modulated by the difference in the interfacial free-energy densities of the phases,^38,46–49^ which in turn depend on the sequence-encoded molecular interactions of the components.^35–37,48^ Phases with high interfacial free-energy densities are expected to be engulfed by those with lower ones,^50^ while some phases form completely separate droplets due to high interfacial tensions.^51^ These observations, together, might suggest that RNA is essential to sustain multiphase condensates, or that a large number of components are needed. But is that the case? To be able to identify the rules governing multiphasicity, here, we focus on systems that are rather simpler, with only two protein components. We develop a molecular-simulations approach that allows us to understand the physicochemical characteristics they must have to form multiphase condensates.

Although one could in principle speculate, based on physical intuition, which pairs of protein sequences might give rise to multiphase architectures, this strategy is likely only feasible for simple amino-acid sequences. Even then, the phase behaviour of multi-component systems with multiple coexisting condensed phases is far more challenging to predict than that of single-component condensates, especially given the complexity and diversity of biologically relevant proteins. Computational approaches, such as genetic algorithms, can help explore the vast size of protein sequence space by automating the design of protein sequence mutations. Broadly speaking, genetic algorithms use mechanisms inspired by biological evolution, such as crossovers and mutations, to optimise properties of a system,^52–54^ and have long been used in optimisation problems in many fields,^55–65^ including those with biological applications such as protein engineering^66–68^ and drug design.^69,70^ Genetic algorithms have recently been applied to evolve protein sequences to (de)stabilise their condensates.^71,72^ However, in order to use genetic algorithms in the context of LLPS, we first need to quantify the protein properties we wish to optimise. Recently, computer simulations have connected features of individual biomolecules to their phase behaviour.^73^ Depending on the question being addressed, models from atomistic^74–80^*via* residue-level^81–87^ to minimal,^80,88–90^ alongside other computational approaches such as predictive algorithms and machine-learning methods,^85–87,91–95^ have all been used with success. Simple models for phase separation of multi-component mixtures and multiphase organisation have also been studied in detail with a combination of simulations and theory.^96–100^

Motivated by these ideas, here we develop an evolutionary algorithm that goes beyond manipulating the stability of condensates and allows us to enforce or inhibit a desired spatial organisation of biomolecules inside multi-component condensates. We couple molecular-dynamics simulations of a residue-resolution coarse-grained protein model that achieves near quantitative agreement with experiments^84^ with a genetic algorithm^71^ to evolve protein sequences towards increasing ‘multiphasicity’, which we define to be the difference in the compositions of the two coexisting phases of a multiphase condensate. The multiphasicity of a condensate increases with the purity of the two coexisting phases. We first demonstrate that we can increase the multiphasicity of a protein mixture using a genetic algorithm with an appropriate fitness function to evolve the amino-acid sequence of one of the two proteins (Section 2.1). We then show that we can design a protein sequence to act as a multiphase partner for some other protein of choice by coevolution (Section 2.2), including proteins of biological relevance such as the low-complexity domain (LCD) of heterogeneous nuclear ribonucleoprotein A1 (hnRNPA1). Finally, we analyse the changes in interaction energies (Section 2.3) and amino-acid patterning (Section 2.4) to probe the factors driving the formation of multilayered condensates.

## 2 Results

### 2.1 Genetic algorithms can improve the separation of two-protein multilayered systems

Genetic algorithms can effectively evolve amino-acid sequences to find mutations that give rise to desired changes in phase behaviour.^71,72^ During the evolution, each protein in a population is associated with a fitness value, as computed with a fitness function, and the population is evolved with local mutations and crossovers between individual sequences towards a fitter – albeit not necessarily the fittest – solution. Rather than determining an optimal solution (however defined), the genetic algorithm follows local gradients in fitness space towards a better solution that is not drastically dissimilar to the starting sequence. The success and efficiency of the genetic algorithm depend on the quality of the fitness function, which should be relatively simple and inexpensive to compute, while at the same time sufficiently complex to act as a suitable proxy for the property being evolved. For simplicity, here we focus on evolving two-component protein condensates towards higher multiphasicity (as defined above). Nonetheless, our approach can readily be generalised to condensates with a larger number of components, which are in any case expected to behave similarly.^45^

**Fig. 1:**
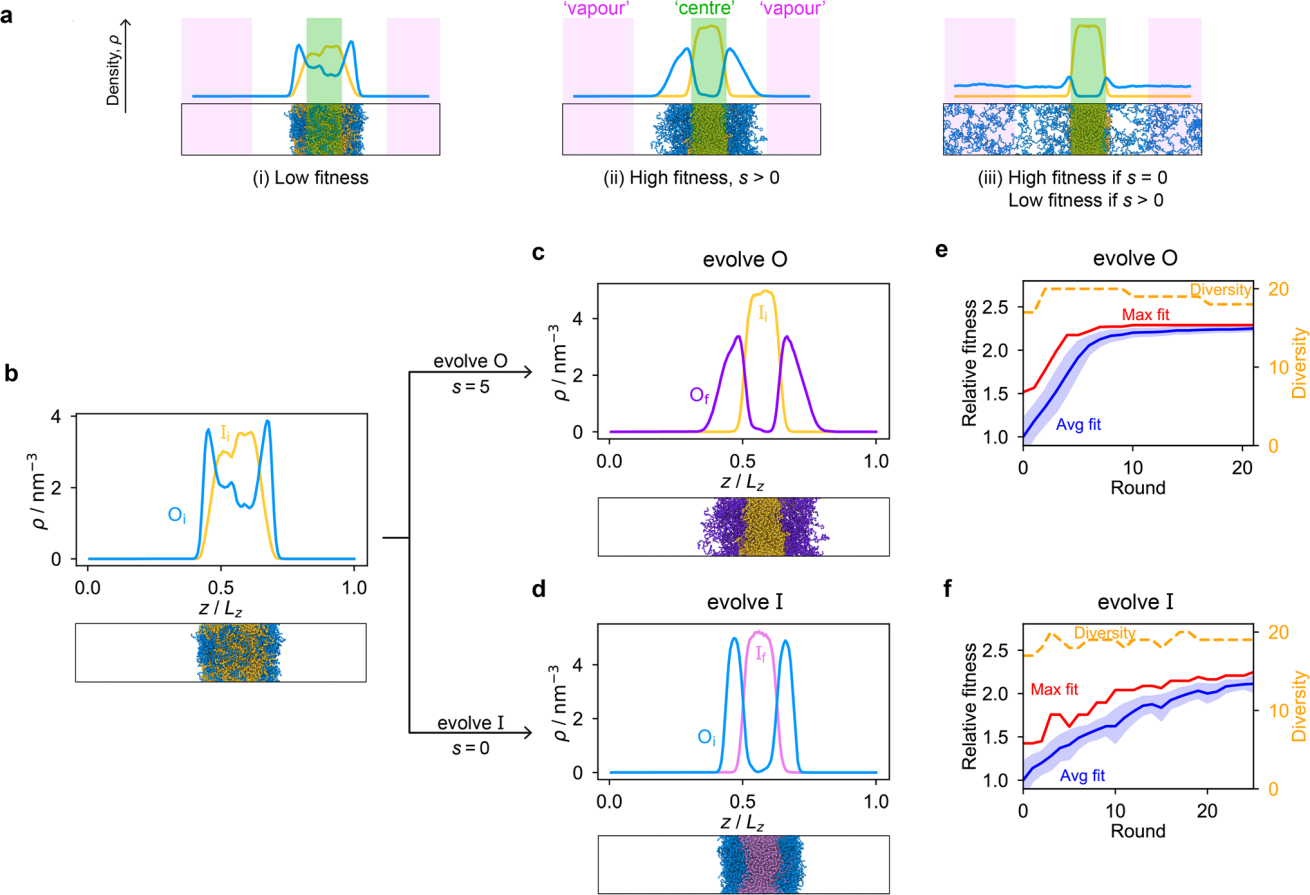
The genetic algorithm can improve multiphasicity. (a) Examples of systems with high and low fitness values. The parameter *s* determines the trade-off between increasing the difference in compositions between the two phases and obtaining two stable liquid-like phases alongside a vapour-like phase. (b) Density profile (number density *ρ* against the long axis of the simulation box) of the initial two-component system considered. Protein I is enriched in the inner layer and protein O in the outer layer. Here O_i_ = (FAFAA)_10_ and I_i_ = F_50_ with random noise added by introducing mutations with probability 0.60 to the latter such that both sequences mix to give a system with low multiphasicity. (c) Density profile of the final evolved system with maximum fitness when protein O is evolved, and (d) when protein I is evolved in separate runs. Snapshots of the corresponding multiphase droplets are provided for each case. (e) and (f) Fitness (relative to the average fitness in round 0) as a function of genetic-algorithm progression. The shaded area corresponds to the standard deviation of the fitness across the population in each round. In both cases, a high population diversity is maintained.

To design our fitness function, we start with the simplest metric of multiphasicity we could conceive for a two-component system: the difference in the number densities between the two different proteins at the centre of the multilayered condensate. This quantity is small when the two-component mixture phase separates into a homogeneous condensate (*i.e.* low multiphasicity) and large when it phase separates into a multilayered condensate with each layer enriched in a different protein (*i.e.* high multiphasicity) [Fig. 1a]. However, if the two-component mixture phase separates into a homogeneous condensate that is depleted of one of the two proteins and a dilute phase that is enriched in the depleted protein, this metric is large even though the multiphasicity is low [Fig. 1a(iii)]. To avoid this, we introduce a second term in the fitness function that penalises the accumulation of either protein in the dilute phase, namely the sum of the number densities of the two components far away from the condensate [eqn (1)], scaled by a weighting parameter *s*. A large *s* might seem desirable to ensure the enrichment of both components inside the condensate; however, when its value is too large, it dominates the fitness function, making the first term irrelevant in magnitude, and can actually favour homogeneous condensates instead. In general, this weighting parameter can be tuned as necessary depending on the specific system of interest [see Fig. S1†].

When evolving a two-component protein system towards increasing multiphasicity, the goal is to obtain a set of mutations to the amino-acid sequences of the two proteins such that the mutated proteins form a condensate with a more segregated multilayered architecture than the starting pair. We refer to the protein enriched in the core of the multilayered condensate as the ‘inner protein’ or ‘protein I’, and the one concentrated in the outer layer as the ‘outer protein’ or ‘protein O’. There are several routes one could take: one could evolve either the inner or the outer sequence in separate evolution runs, or even evolve both sequences, simultaneously or alternately, in the same run. In our evolution runs, we evolve either the inner sequence or the outer sequence whilst keeping the other sequence unchanged in order to simplify the subsequent analysis of driving forces. To evolve the sequence, we apply the genetic algorithm as described above (and in the Methods section), computing a sequence's fitness by performing direct-coexistence molecular-dynamics simulations of the two-component mixture in a slab geometry using our residue-resolution coarse-grained model, Mpipi,^84^ at a fixed temperature.

As a preliminary test of our approach, we first consider a mixture of (FAFAA)_10_ and F_50_ and add sufficient mutations (*i.e.* random noise) to the latter to ensure that the initial condensate has a low fitness. The mutations are added in a similar way to how the initial population of sequences is generated in the genetic algorithm (Methods), but with a replacement probability of 0.60. In this initial state, (FAFAA)_10_ is slightly enriched at the interface and is deemed the outer sequence, but there is an appreciable degree of mixing with the inner sequence. We then perform two separate evolution runs, evolving (a) the inner sequence whilst keeping the outer unchanged, and (b) the outer sequence whilst keeping the inner sequence unchanged. A comparison of the density profiles of the initial system to that of the final systems with the maximum fitness [Fig. 1b–d] confirms that our fitness function can successfully guide the initial system towards increasing multiphasicity in both cases. When the inner sequence is evolved, a stable multilayered condensate can be obtained with a weighting parameter *s* = 0 used in the fitness function; by contrast, when evolving the outer sequence, the final result is sensitive to the value of the parameter *s* and *s* > 0 must be used. We show in Fig. S1† the final evolved systems obtained with different values of *s*. Additionally, the genetic-algorithm progressions in each case are depicted [Fig. 1e and f]. We plot the mean fitness of the population (blue curve), the fitness of the fittest individual (red curve) and the number of distinct sequences in the population (*i.e.* its ‘diversity’; yellow curve). Finally, we show the results for an evolution run starting from a different initial system with different sequences in Fig. S8a and b,† demonstrating that the behaviour observed is not sensitive to the initial sequence choice.

### 2.2 Multilayered condensates can be designed by coevolving a partner protein sequence alongside a protein of interest

**Fig. 2:**
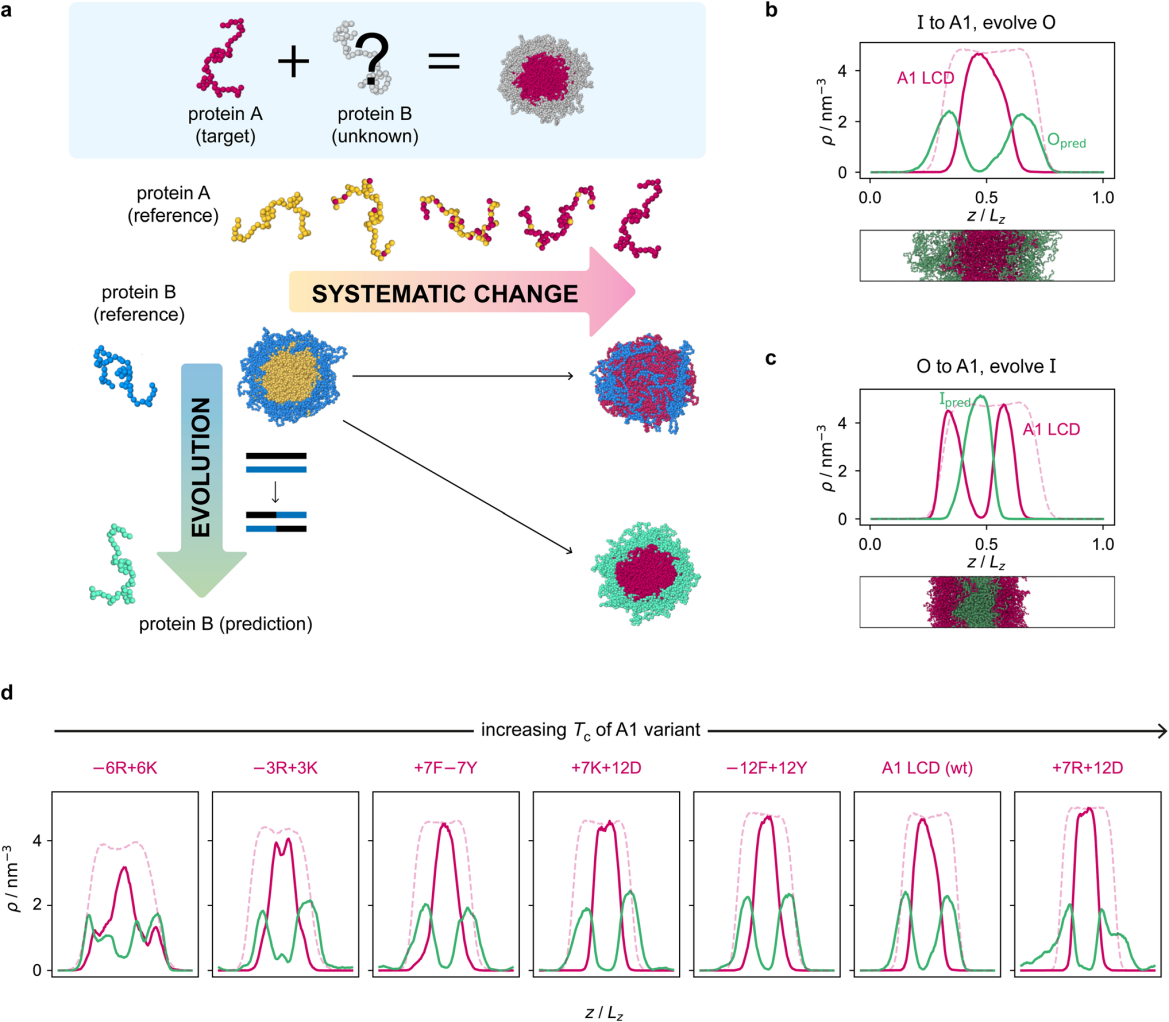
Coevolution of a multilayered condensate partner for A1 LCD. (a) Illustration of the coevolution approach. Starting from an initial two-component reference system with high multiphasicity, we systematically change one sequence to the predetermined target protein (yellow to pink), while simultaneously evolving the other sequence (blue to green) to predict a partner sequence that forms a multilayered condensate with the target protein. Spherical droplets are shown for clarity; in direct-coexistence simulations, we use a slab geometry instead. (b) Density profile of the final evolved system with maximum fitness from the coevolution run where A1 LCD is designed to be at the centre and (c) where A1 LCD is designed to be on the outside of the condensate. The pink dashed line is the density profile of a single-component system of A1 LCD equilibrated at the same temperature (250 K). (d) Density profiles of the final evolved systems with maximum fitness from the coevolution runs with different variants of A1 LCD as the inner sequence. The densities of the A1 variant and the predicted outer partner sequence are plotted in pink and green, respectively. The pink dashed lines are the density profiles of a single-component system of the corresponding A1 variant equilibrated at the same temperature. The A1 variants are arranged in order of increasing upper critical solution temperature.

Armed with an effective fitness function for multiphasicity, we next set out to use our genetic algorithm to guide the design of partner proteins that result in multilayered condensates alongside known phase-separating proteins of interest (*e.g.* a naturally occurring protein, such as hnRNPA1 LCD used below). There are many challenges involved in designing a partner protein for this purpose, such as ensuring that it phase separates under similar experimental conditions to the protein of interest (*e.g.* salt, pH, and temperature), and that it establishes suitable associative interactions with the protein of interest to form a single multilayered condensate. Thus, to facilitate convergence in this more complex scenario, we start our coevolution approach [Fig. 2a] from an initial reference system of two proteins, both different from our protein of interest, which phase separates into a multilayered structure with a high degree of multiphasicity. The initial reference systems used in the coevolution runs are designed using simple generic sequences of amino acids based on knowledge from previous experimental and theoretical studies showing that immiscible phases form when interaction strengths between their components are sufficiently different.^35,36,45,48,49,101^ In particular, we choose the inner sequence to have a high aromatic sticker content [*e.g.* F_135_ or Y_135_; see below], since aromatic residues exhibit strong favourable interactions. For the outer sequence, we choose a simple sequence that combines some sticker residues with spacer residues [*e.g.* (FAFAA)_10_], so that the overall interaction is less strong compared to the inner sequence. To arrive at our protein of interest, we then systematically mutate one of the two reference sequences throughout the coevolution run. These systematic mutations are done gradually, once every 5 rounds, by randomly changing ∼10% of the residues of this protein selected from those that have yet to be changed to the amino acid of the target protein. Simultaneously, we evolve the other sequence with the genetic algorithm using our fitness function [eqn (1)].

Since only a small proportion of the residues in the sequence that is systematically changed are modified each time, multiphasicity can be maintained at least to some degree throughout the process. This procedure ensures that there is a gradient of the fitness in sequence space in the direction of increasing multiphasicity which the genetic algorithm can evolve towards at every round during the coevolution. If one started from the reference multilayered system and changed one of the sequences to the target sequence in one go, this may result in full mixing of the two components within a homogeneous liquid-like phase and hence the loss of multiphasicity altogether. From combinatorics, there are numerous sequences that can form a homogeneous condensate with the target sequence; using the genetic algorithm starting from a fully mixed state would therefore be inefficient, as the initial random search for possible mutations that result in multiphasicity would be slow before there is a gradient in sequence space towards increasing multiphasicity that can be exploited by the genetic algorithm.

Of course in some cases, changing one sequence in the reference system to the target protein may still give a multilayered system, albeit likely with a lower degree of multiphasicity. Alternatively, one may also be able to propose, based on physical intuition and understanding of the intermolecular interactions that give rise to the formation of multiphase condensates, a protein sequence that can form a multilayered condensate with the target protein. In such situations, it is possible to use the genetic algorithm directly to evolve the system towards an increasing degree of multiphasicity, as discussed in Section 2.1. However, importantly, the coevolution approach we have outlined would find a possible solution much more efficiently even if an initial multilayered system with the target protein is unknown and difficult to predict by hand.

Here, to demonstrate the robustness of the coevolution approach, we select the initial reference systems and target sequences in the coevolution runs such that making the systematic change directly in one go results in complete mixing to give a single homogeneous liquid-like phase [*cf.* Fig. S19a†]. We show the results of the coevolution approach tested on systems with simple generic sequences in Section S12.

#### 2.2.1 A multiphasic partner can be found for hnRNPA1 LCD and its variants

To demonstrate how the coevolution approach can be used to design multiphase condensates containing naturally occurring phase-separating proteins, we turn our attention to the LCD of hnRNPA1 (denoted here as A1 LCD). We first focus on designing a multilayered condensate with A1 LCD concentrated at the centre, and below we look at the converse case. To predict the partner sequence that forms a multilayered condensate with A1 LCD at the centre, we start our procedure with a mixture of I = F_135_ and O = (FAFAA)_10_, and then systematically change the inner protein to A1 LCD whilst evolving the outer protein using the genetic algorithm. We choose the initial inner protein such that its length is the same as that of A1 LCD. During the first 45 rounds of the coevolution procedure, the residues of the inner protein F_135_ are systematically and gradually changed to those of A1 LCD [Fig. S2e†], whilst the outer protein (FAFAA)_10_ is evolved. We continue the genetic algorithm on the outer protein for an additional 20 rounds to increase the degree of multiphasicity of the system further. We show the density profiles and snapshots of the initial system and the final evolved system at the end of the coevolution run in Fig. S2b and c;† the final system exhibits two liquid-like phases of different composition with A1 LCD enriched in the centre, demonstrating that the coevolution approach is able to predict a partner sequence that forms a multilayered condensate with A1 LCD.

The genetic-algorithm progression of this coevolution run is shown in Fig. S2d.† The average fitness and the maximum fitness suddenly decrease in rounds where systematic changes are made and the fitness of the entire population is recalculated. Although the outer sequence is evolved using the genetic algorithm for a considerable number of rounds after the inner sequence has been completely changed to A1 LCD, the maximum fitness does not improve in these rounds, suggesting that we have reached a local maximum in the fitness function. Changing the starting sequence of the protein being evolved does not appear to offer sufficient flexibility to support a higher degree of multiphasicity. We hypothesise that a higher multiphasicity might be achieved by increasing the length of the partner sequence that is evolved, since more sequence variations are possible with a longer sequence. To test this idea, we increase the length of the outer protein from 50 to 100 residues, but keeping the total number of protein residues unchanged, and repeat our coevolution procedure. Specifically, we start the coevolution from a mixture of I = F_135_ and O = (FAFAA)_20_, and then systematically and gradually change the inner protein to A1 LCD whilst evolving the outer sequence [Fig. S4a†]. The density profile of the final evolved system with the longer partner sequence is shown in Fig. 2b [see also Fig. S6b and S5a†]. As hypothesised, the degree of multiphasicity of the final system is considerably improved with a longer protein partner, likely, as speculated, because of the greater flexibility in sequence choice with a longer protein.

In principle, the resulting partner sequences obtained from the coevolution run depend on the identity of the two proteins in the initial reference system, and it is not immediately obvious how to choose the reference system sensibly. Indeed, our work highlights that the solution to this problem is not unique and multiple different partner sequences can form diverse multilayered condensates with a specific target protein of interest. If we wish to find a possible solution, rather than a specific one, starting from any reference multilayered system should be feasible. To demonstrate this, we repeat the coevolution run starting from a different reference system with different protein sequences, namely a mixture of O = N_100_ and I = Y_135_. The latter, at the centre of the multilayered condensate, is then systematically changed to A1 LCD, while the outer protein is evolved using the genetic algorithm. Density profiles [Fig. S3b and c†] confirm that the coevolution approach is again successful; of course, unsurprisingly, the final evolved partner sequence is considerably different from before, since we expect it to retain at least some features from the initial reference sequence.

We now test the ability of our coevolution algorithm to predict the amino-acid sequence of a protein partner that forms a multilayered condensate with A1 LCD concentrated towards the interface of the condensate. To do so, we construct a reference multilayered system of I = (FAFAA)_20_ and O = (FIQII)_27_, but now we change the outer protein systematically and gradually to A1 LCD whilst evolving the inner one. As desired, we show [Fig. 2c] that the system forms a multilayered condensate with A1 LCD towards the interface [see also Fig. S6c and S5b† for further details].

Finally, to demonstrate the robustness of the approach to the target protein sequence, and to allow us to investigate if there are any overarching governing principles of multiphasicity that we can identify, we repeat the coevolution approach to find partner sequences for different variants of A1 LCD. In these cases, we choose the final multilayered condensates to have the A1 LCD variant concentrated in the centre. The phase diagrams of a set of A1 LCD variants have recently been determined both experimentally^102^ and computationally using the same coarse-grained model that we have used in this work.^84^ We consider sequences with similar, higher and lower upper critical solution temperatures compared to the wild type (WT), ensuring that sequences with distinct features are represented. In particular, we focus on two aromatic variants, +7F−7Y and −12F+12Y, two mixed-charged variants, +7R+12D and +7K+12D, and two arginine–lysine variants, −6R+6K and −3R+3K. As for the WT, we start coevolution runs from a mixture of I = F_135_ and O = (FAFAA)_20_. We show the density profiles of the final evolved systems in Fig. 2d and the corresponding genetic-algorithm progressions in Fig. S5c–h.† The coevolution approach successfully predicts a suitable partner sequence for each A1 variant. The overall trends in the change in fitness are similar for all of the variants, although the final systems have varying degrees of multiphasicity. In particular, the final evolved systems with the −6R+6K and −3R+3K variants, which have considerably lower critical temperatures than the WT, are less well separated. We speculate that this result can be improved with a longer partner sequence, as we have shown for the WT.

### 2.3 Multilayered condensates are driven by the difference in component interaction strengths

Predicting a partner sequence to design multilayered condensates containing a certain target sequence is of practical importance. However, molecular simulations allow us to go one step further and understand the underlying physical and molecular driving forces for the observed behaviours. To identify which properties are important for multiphasicity, we analyse the changes in the composition and patterning of the evolved sequence in the evolution runs with generic sequences, where one component remains unchanged throughout, and in the coevolution runs with systematic changes of one component to A1 LCD.

**Fig. 3:**
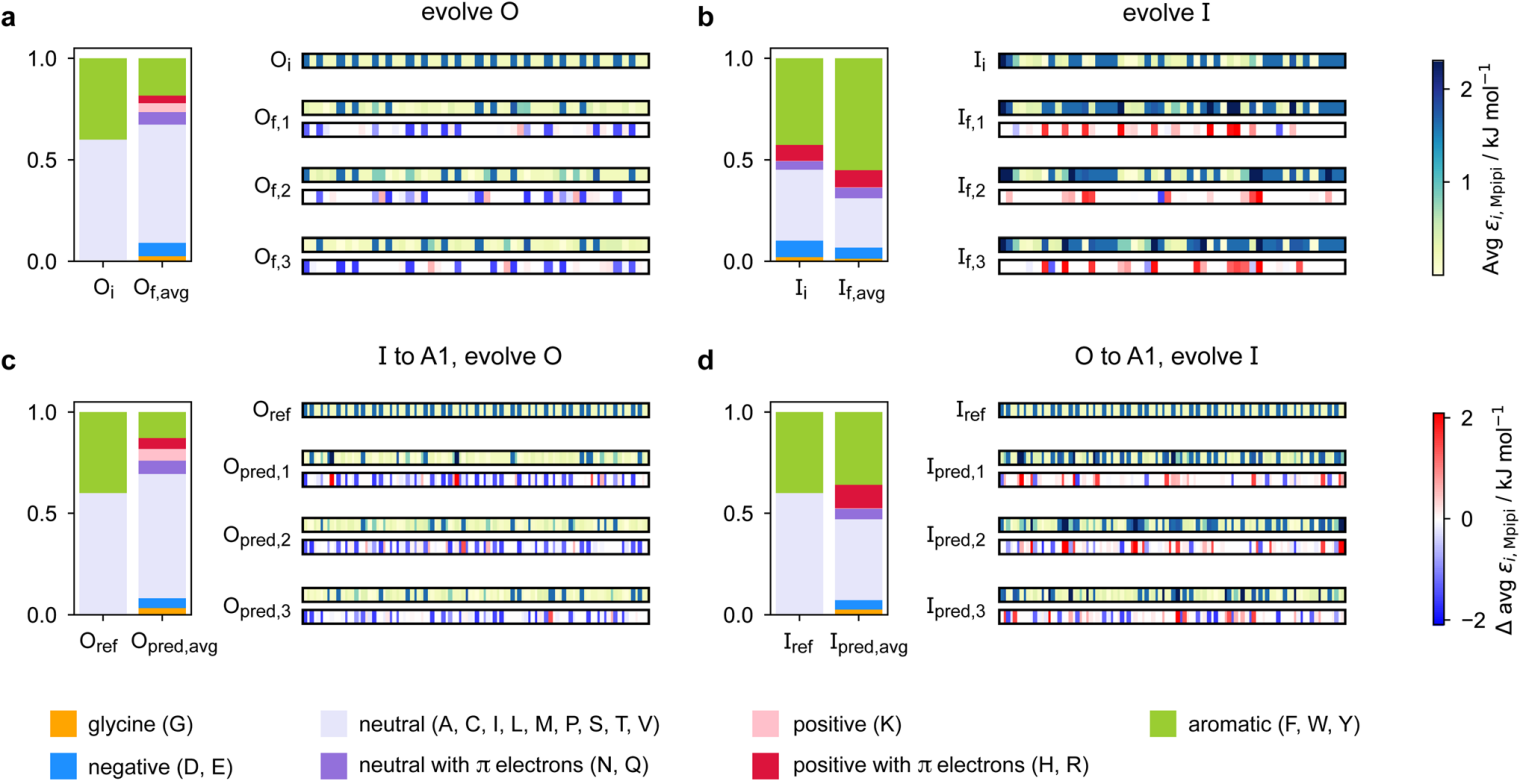
Amino-acid composition and patterning on evolution. Comparison of the amino-acid composition and sequence patterning between the initial and final evolved sequences with maximum fitness in the evolution runs where (a) the outer sequence is evolved and the inner sequence is kept unchanged and (b) the inner sequence is evolved and the outer sequence is kept unchanged; and the coevolution runs where A1 LCD is designed to be (c) the inner sequence and (d) the outer sequence in the final multilayered system. For each case, we show the composition of the initial sequence and the final evolved sequence averaged across three independent runs. To illustrate the final evolved sequences in three independent runs in each case, we plot for each residue *i* along the sequence the absolute value of and the change in *ε*_*i*,Mpipi_ compared to the residue in that position in the initial sequence. The value of *ε*_*i*,Mpipi_ estimates the interaction strength of the residue in the coarse-grained model.

We summarise the changes in the amino-acid composition of the evolved sequences in Fig. 3. In the evolution runs with generic sequences where the outer sequence is evolved while the inner sequence is kept unchanged, the amino-acid composition of the evolved sequence changes to favour fewer aromatic residues [Fig. 3a]. By contrast, when the inner sequence is evolved while the outer sequence is kept unchanged, evolution favours a higher number of aromatics [Fig. 3b]. These observations suggest that within multilayered condensates, when other features are kept constant (*e.g.* protein charge, disorder and length), protein sequences with a higher aromatic content are likely to cluster towards the centre. The mean interaction strengths amongst amino acids change across the evolution runs; we use the parameter *ε*_*i*,Mpipi_ of the Mpipi model [see the Methods section] to estimate these changes. The average of *ε*_*i*,Mpipi_ across all residues in the evolved sequence decreases over the course of the genetic-algorithm run when the outer sequence is evolved [Fig. S7a†], but increases when the inner sequence is evolved [Fig. S7b†]. By comparing the final evolved sequences with maximum fitness across three independent evolution runs [Fig. 3], we note that residues become more strongly interacting on average when the inner sequence is evolved, and conversely, more weakly interacting when the outer sequence is evolved. As evidenced by Fig. 3, there are numerous ways of achieving such a change in interaction strengths, and the solution to this optimisation problem is unsurprisingly not unique. This is because for a given predetermined sequence, from combinatorics, there should exist multiple different protein sequences that can form a multilayered condensate with it, *i.e.* many sequences are similarly fit and the corresponding well in the fitness landscape is broad. This important observation puts forward the idea that the formation of multiphase condensates is a general phenomenon requiring only a generic set of interaction rules governed by the overall chemistry of the functional groups involved (*e.g.* π-rich, charged, or hydrophilic), rather than highly sequence-specific features.

When designing multiphase condensates that have the phase of A1 LCD proteins or its variants at the centre, we observe that the proportion of aromatic residues in the evolved partner protein decreases [Fig. 3c, S7c and S14a†]. The more strongly interacting residues are preferentially replaced by less strongly interacting ones throughout the evolved partner protein sequence. This change in composition of the evolved sequence is similar to the trend we observe in the evolution run with generic sequences where the outer sequence is evolved and the inner sequence is kept constant. The final evolved sequences in the coevolution runs with the different A1 variants are also similar in terms of the change in composition [Fig. S13a†], even though we selected variants with different features. Besides a decrease in the proportion of aromatic residues, we also observe that there is a slight upward trend in the average net charge per residue across the sequences in the population [Fig. S13b†]. The effect of increasing net charge per residue weakening the tendency to form condensates due to increasing repulsion or promoting solvation has been investigated by Bremer *et al.*^102^ This may be another mechanism to decrease the stability of the condensates formed by the evolved outer protein in this case, although we note that the increase in net charge per residue is small. Altogether, the main driving force for the evolution towards increasing multiphasicity of condensates with A1 LCD at the centre is the decrease in the average interaction strength of the outer sequence. However, for the case where A1 LCD is designed to be on the outside of the multilayered condensate and the inner sequence is evolved, the proportion of aromatic residues in the final evolved sequences is similar to the initial inner sequence and it is less clear whether the residues become more or less strongly interacting throughout the sequence [Fig. 3d and S7d†]. This is not entirely surprising, since in the coevolution runs the two sequences in the system are being changed and evolved simultaneously, so we cannot necessarily expect the same trends as when only one sequence is evolved.

**Fig. 4:**
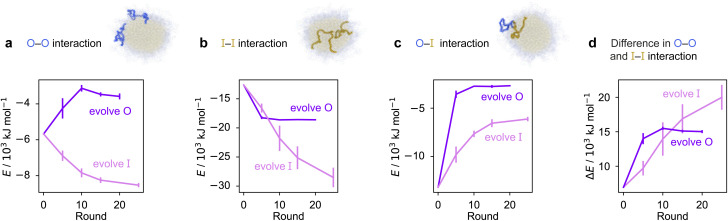
Homo- and heterotypic interaction strengths control multiphasicity. The change in the interaction energies of intermolecular homotypic interactions between proteins enriched in the (a) outer and (b) inner layers, (c) heterotypic interactions between the two proteins and (d) the difference between the outer–outer and inner–inner homotypic interactions for the system with the fittest individual. Purple curves correspond to the system where the outer protein was evolved and the inner was unchanged, and pink curves correspond to the converse. Error bars correspond to the standard deviation across three independent runs. Spherical droplets are shown for clarity; we use a slab geometry in direct-coexistence simulations.

To rationalise why the compositional changes we observe in the evolved sequences favour multiphasicity, we compute the strengths of homotypic and heterotypic interactions between proteins forming the inner and the outer phases, and their changes throughout the evolution runs [Fig. 4]. Overall, our analyses reveal that the formation of two-component multilayered condensates depends on three crucial requirements. First, larger differences in the strengths of homotypic interactions of the different species (*i.e.* inner–inner *versus* outer–outer) favour demixing of the components into separate phases [Fig. 4d], similar to what has been observed in modelling work by Jacobs *et al.*^45^ and Feric *et al.*,^38^ as well as experimental work with minimal systems by Fisher *et al.*^36^ Second, the proteins that establish the stronger homotypic interactions form the inner phase of the multilayered condensate. In other words, the inner–inner interaction is always the strongest, likely because such an arrangement guarantees saturation of binding sites that can form the most energetically favourable interactions. Third, the strength of heterotypic interactions should lie on a critical ‘sweet spot’: small enough to favour demixing into separate phases, but sufficiently large (*i.e.* comparable with the weaker outer–outer homotypic interactions) to keep the separate phases coexisting inside the same condensate.

Our evolution and co-evolution algorithms induce changes in the homotypic and heterotypic interactions across the evolution that depend on the properties of the starting condensates and which of the proteins is being evolved. For the evolution runs with generic sequences where one sequence is evolved and the other kept unchanged, our starting system exhibits relatively modest strengths for both the inner–inner and outer–outer homotypic interactions, and comparatively strong heterotypic interactions. These heterotypic interactions become weaker throughout the evolution runs when either the inner or the outer protein sequence is evolved [Fig. 4c]. The inner–inner homotypic interactions also become stronger in both cases, although the strengthening is rather more pronounced when the inner protein is being evolved [Fig. 4b]. This is expected when the two coexisting condensed phases become more pure and the two components become less well mixed.^103,104^ This substantial strengthening in inner–inner interactions obtained when evolving the inner sequence indirectly results in the outer–outer interactions also becoming stronger as both phases become purer, even though the outer sequence is itself kept unchanged. By contrast, when the outer protein is evolved, the outer–outer homotypic interactions weaken even as the outer phase becomes purer [Fig. 4a]. Nevertheless, in both cases, we observe that the balance of interactions converges to the same behaviour across evolutionary runs: the difference in homotypic interactions becomes larger, while the heterotypic interactions become weaker and comparable in strength to the outer–outer interactions. The trends we observe for the changes in composition and interaction energies seem to be robust to the choice of the initial system [Fig. S8d–f and S9†]. The interaction energies in our co-evolved multiphase condensates with A1 LCD also meet the criteria we describe above [Fig. S11†]. That is, multiphasicity emerges for systems with sufficiently different homotypic interactions, where the inner–inner interactions are strongest and heterotypic interactions are small but comparable to the outer–outer interactions.

Previous work has shown that the excluded volume interactions of the residues, particularly of spacers, are important for multiphasicity.^99,105^ In the evolution run with generic sequences where the inner sequence is evolved and the outer sequence is kept unchanged, we note that the average value of *σ*_*i*,Mpipi_ increases as the fitness function increases [Fig. S10†], and this may in part be related to the suggested increase in compositional demixing with increasing excluded volume. However, since only amino-acid residues are changed in the evolution runs and they all have relatively similar sizes, the changes in *σ*_*i*,Mpipi_ are much smaller than studied in previous work, and residues with larger *σ*_*i*,Mpipi_ may be favoured by the increase in interactions arising from the larger cutoff rather than the excluded volume itself.^71^

Finally, we compute the interfacial free-energy densities for the liquid–vapour interface for bulk A1 LCD and its final coevolved proteins [see the Methods section and Fig. S12†]. These results are shown in Table 1 and confirm the expectation that the protein with the largest surface tension with its vapour is most likely to be at the centre^46^ of the multilayered condensate. By computing the interfacial free-energy density at several different temperatures [Fig. S12†], we can extract the interfacial entropies^106^ and in turn the interfacial energies; these are also shown in Table 1. The formation of the interface is energetically unfavourable; although it is in principle entropically favourable, since molecules in the liquid-like phase can gain considerable translational entropy at the interface, this contribution is relatively small. The difference in homotypic energies between species therefore also dominates the thermodynamic favourability of interface formation and determines the ordering of the layers in a multilayered condensate.

**Table 1:** Interfacial thermodynamic parameters^a^

	*γ*/mJ m^−2^	*S*_int_/J m^−2^ K^−1^	*E*_int_/mJ m^−2^
I_pred_	3.4(3)	17.1(1)	7.6(3)
A1 LCD	0.9(4)	9.4(7)	3.2(2)
O_pred_	0.03(18)	6.3(4)	1.61(8)

a Interfacial free-energy density *γ* at 250 K, interfacial entropy density *S*_int_ and interfacial energy density *E*_int_ for A1 LCD and its final coevolved proteins with maximum fitness when (i) the evolved protein is on the inside of the condensate (I_pred_) and A1 LCD is on the outside and (ii) the evolved protein is on the outside of the condensate (O_pred_) and A1 LCD is on the inside. Errors in brackets apply to the least significant digit and give the standard errors of the fitting parameters [Fig. S12].

Overall, our analyses explain why residues that increase the difference in interaction strengths between the two sequences improve multiphasicity. These results support previous studies which found that multiple immiscible phases are formed when there is a sufficient difference in interaction strengths between the components in the two phases.^35,36,38,45,48,49,101^

### 2.4 Sequence patterning is only sometimes important for multiphasicity

The patterning of interacting amino-acid groups plays an important role in determining the phase behaviour of intrinsically disordered proteins (IDPs).^71,107^ For example, the range of stability of A1 LCD condensates was shown to depend on the number and patterning of aromatic residues, which act as stickers in the ‘stickers-and-spacers’ framework.^107,108^ More uniform distributions of stickers were found to promote the phase separation of A1 LCD and to decrease the propensity to form aggregates.^107^ However, in our initial evolution runs with generic sequences and coevolution runs with A1 LCD, we have shown that the final evolved sequences in independent repeats of the same run, despite having similar overall compositions, can differ considerably in terms of the patterning of the more strongly interacting sticker residues.

To investigate the importance of the patterning of different residues in determining the degree of multiphasicity of these two-component systems, we shuffle the final evolved sequence with maximum fitness by rearranging residues of interest (*e.g.* stickers or spacers) whilst keeping the overall composition of the sequence unchanged, and compute the density profiles and fitness of shuffled sequences to examine the effect of shuffling on phase behaviour. In our analyses, we consider as stickers all the aromatic residues (F, Y, and W), the neutral residues with π electrons in the side chain (N and Q) and arginine (R), and the remaining residues as spacers.

We first do this for the final fittest systems resulting from the evolution runs with generic sequences (without A1 LCD) where one sequence is evolved and the other is kept unchanged. Unexpectedly, in these systems, when we shuffle (in multiple different ways) the evolved sequences stemming from runs where one sequence is evolved and the other is kept unchanged, the multiphasicity and hence the fitness are not notably altered [Fig. S15a and b†]. Even in the extreme cases where the evolved sequence is sorted such that the residues are rearranged in order of increasing *ε*_*i*,Mpipi_ values, with all the strongly interacting sticker residues clustered together at one end of the protein, the multilayered structure was still maintained, albeit with a drop in fitness indicating a lower degree of multiphasicity in some cases. This would suggest that for these sequences, the patterning of the stickers and spacers has a minimal effect on the formation of the two coexisting phases, and that it is only the overall composition of the sequence that determines whether the two proteins will mix into one homogeneous phase.

**Fig. 5:**
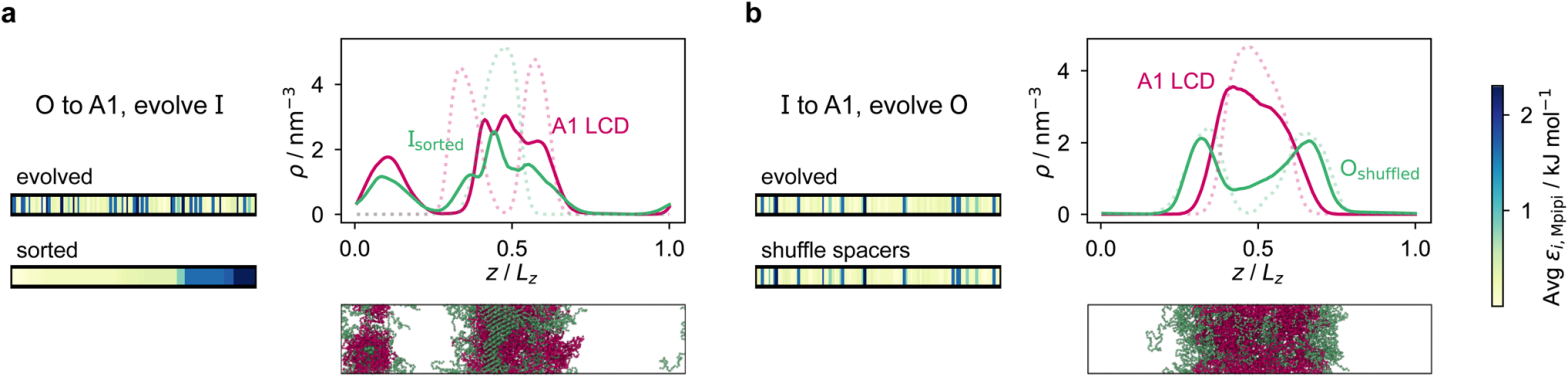
Effect of sequence patterning on the formation of multilayered condensates. (a) Density profile of the final evolved system with maximum fitness in the coevolution run where A1 LCD is designed as the outer sequence, but with the residues in the final evolved sequence I_pred_ rearranged in terms of increasing *ε*_*i*,Mpipi_. The sorted sequence with all the strongly interacting residues clustered at one end results in rather different phase behaviour. (b) Density profile of the final evolved system with maximum fitness in the coevolution run where A1 LCD is designed as the inner sequence, but with spacer residues (see the text) in the final evolved sequence O_pred_ shuffled randomly. The sequence with the spacers shuffled resulted in a decrease in multiphasicity compared to that of the original evolved sequence predicted by the coevolution approach. Dotted lines represent the density profile of the original evolved system with the unshuffled partner sequence.

However, for the coevolution runs with A1 LCD, we do see patterning-dependent behaviour: a sorted sequence, with stickers at the ends of the protein molecules, results in rather different phase behaviour [Fig. 5a] compared to the original coevolved sequence [Fig. 2c]: in the sorted case, the sticker-rich ends interact so strongly that they form a locally crystalline structure. Interestingly, for the case where A1 LCD is at the centre of the multilayered condensate, shuffling just the positions of the spacers in the evolved sequence results in a lower degree of multiphasicity [Fig. 5b] relative to the system with the original evolved sequence [Fig. 2b]. The heterotypic interactions become stronger and the difference in homotypic interactions of the two sequences decreases after shuffling, consistent with the lower degree of multiphasicity observed. The fact that different spacers do not give rise to identical phase behaviour has recently been investigated by Bremer *et al.*,^102^ and similarly, in this case, we find that it is not just the distribution of stickers *versus* spacers that is important, but also both the identity of the spacers and their arrangement along the sequence.

Although in some cases patterning of amino-acid residues does not affect the phase behaviour much, it does in others. It would be helpful to anticipate the conditions where patterning is likely to be important. Our tests suggest that if the partner protein's sequence is repetitive with low compositional diversity, the relevant interactions can occur anywhere along the chain, reducing the need for a particular patterning of interactions to maintain phase separation. For example, for the case with generic sequences where we evolve the outer sequence and the inner protein is I = (FAFAA)_10_, which is highly repetitive, the multiphasicity is unaffected by shuffling or sorting the outer protein [Fig. S15a†]; however, if we sort the inner sequence to give I = F_20_A_30_, thereby removing the repetition while maintaining the overall composition, this results in a substantial loss of multiphasicity [Fig. S16†]. By contrast, the protein partner of the analogue where the inner sequence is evolved is not especially repetitive [see protein I_i_ of Fig. 3b], but patterning is nevertheless not especially important [Fig. S15b†]. Another obvious difference between the sequences investigated is their length, and it may appear that with shorter proteins (such as those investigated in Fig. S15a and b†), all relevant residues are spatially sufficiently close that the same interactions dominate irrespective of their precise position in the sequence. However, the behaviour of systems where A1 LCD is partnered with a 50-residue strand (*cf.* Fig. S2†) is almost identical to the case of 100-residue strands shown in Fig. 5, S15c and d,† suggesting that the difference in length alone is not sufficient to rationalise the difference in behaviour.

The phase behaviour of IDPs has been observed to be affected by the patterning of not only aromatic residues, but also charged ones.^99,109–112^ The role of charge patterning has been investigated with simpler polymer models containing charged segments in the context of demixing in both one-component^111,113^ and two-component^97–99^ systems. In the latter, a large mismatch in charge patterning between the two sequences has been found to favour compositional demixing.^97–99^ We investigate the effect of charge patterning mismatch on the degree of multiphasicity in the shuffled systems with the system where shuffling amino-acid residues resulted in the largest variation in phase behaviour, namely the system where A1 LCD is concentrated at the centre. We show the variation of the sequence charge decoration (SCD), an order parameter quantifying charge patterning [see Section S10], in Fig. S17;† however, we observe little correlation between the mismatch in charge patterning and the fitness. The reason for the insensitivity to charge patterning in this case may simply be that phase separation is not principally charge-driven in the systems we have considered, compared to previous work where the polymers considered were entirely made up of charged residues. We expect that, if the proportion of charged residues in the relevant proteins was larger, there may be a stronger correlation between demixing (as quantified by the fitness function) and the charge pattern mismatch.

Since it is difficult to know *a priori* when the patterning of residues is likely to affect the phase behaviour of multilayered condensates, the use of a genetic algorithm and the coevolution approach as a predictive tool, where any relevant patterning is optimised alongside the interaction strengths, is especially attractive.

## 3 Discussion

We have developed a computational approach to design multi-component multilayered condensates that contain a target protein of interest. Our approach integrates a genetic algorithm, anchored in an innovative fitness function for automated evolution of multiphasicity, with our near-quantitative residue-resolution coarse-grained protein model, Mpipi.^84^ We demonstrate the utility of our approach in a biological context by applying it to predict different protein partners capable of forming two-component multiphase condensates when mixed with A1 LCD or its variants. We show that our method can be adapted to produce condensates that concentrate the protein of interest (*e.g.* A1 LCD) either at the centre of the multilayered condensate or in the outer layer, as desired.

In addition to enabling the design of multiphase condensates, our approach helps uncover the biophysical mechanisms that drive the formation of complex multilayered organisations. In all cases, we find that multiphasicity in multi-component protein systems is favoured if the difference between homotypic interactions among different components is large, and the strength of heterotypic interactions is small but comparable with that of the weaker homotypic interactions in the mixture. In a two-component system, proteins that establish stronger homotypic interactions are concentrated at the core of the multiphase condensate, as saturating their bonds enhances the overall enthalpic gain for condensate formation. Similarly, the outer layer of the multiphase condensate is formed by the proteins that establish weaker homotypic interactions, as this reduces the overall interfacial free-energy density of the two-component system. Although the specific predicted partner sequences can differ across independent (co)evolution runs and several sequences can have similar fitness values, these general trends in interaction energies remain consistent in the cases we have tested, suggesting that the rules we have identified may be universal in driving the formation of multiphase condensates. The diversity of partner sequences obtained may also suggest that a given protein can form multiphase condensates with a wide range of different partners, rather than exclusively with a unique complementary sequence: multiphasic organisation thus appears to be a robust property of multi-component condensates.

Since the genetic algorithm is coupled to a residue-resolution coarse-grained model for proteins, the accuracy of our predictions is contingent on that of the model. Reassuringly, we have previously demonstrated that Mpipi reproduces the experimental phase diagrams of A1 LCD and its variants with near-quantitative accuracy, achieves excellent agreement with experiments probing the phase behaviour of naturally occurring proteins (*i.e.* FUS, Ddx4, LAF-1 and their variants) and of polyR/polyK/polyU mixtures, and predicts the experimental radii of gyration of a large set of IDPs with high accuracy.^84^ We are therefore hopeful that the predictions of our approach will be robust against experimental validation, which is an essential next step. An important factor to consider for such validation is that the specific amino-acid sequences we predict to from multiphase condensates are only applicable at the fixed temperature and salt concentration at which the simulations are run; however, these could of course be changed to design multilayered condensates that are stable under different conditions. One possible limitation of using residue-resolution coarse-grained models like Mpipi is that they are typically unable to consider the emergence of secondary or tertiary structural transitions from specific changes in amino-acid sequence. In this regard, our evolutionary approach is flexible enough to incorporate knowledge-based constraints to bypass selected patterns of amino-acid sequences known to favour, for instance, the folding of protein regions into α-helices or β-sheets in specific contexts, or limit the number of certain residues such as cysteine, which forms disulphide bridges. Our approach can also easily be modified to consider special requirements for each protein system by introducing further constraints in the algorithm: for instance to introduce tailored replacement probabilities and outcomes for different residues (*e.g.* to limit mutations to stickers, only allow mutations of charged to charged residues, or enforce a given pattern of aromatics) or protein regions (*e.g.* to avoid mutations at the N-terminus or to favour the concentration of aromatics at the centre) when proposing mutations.

While we have investigated multiphase condensates comprising only of two protein components, our evolutionary approach is transferable to multi-component systems with a larger number of components, and can also easily be extended to study the effect of RNA or post-translational modifications. In turn, our method expands the repertoire of tools available to gain molecular insight into LLPS in complex biological cellular functions. Our approach therefore presents new opportunities for designing multilayered condensates, probing more closely the underlying physicochemical factors that lead to their formation and, ultimately, deciphering the missing links to their function inside cells.

## 4 Methods

### 4.1 Genetic algorithm and fitness function

The basic framework of our genetic-algorithm approach is very simple:

(1) Choose an initial system with a degree of multiphasicity, such as one of the minimal systems we have outlined.

(2) Choose a target protein for which to design a partner.

(3) Decide whether the target protein should be on the inside or the outside of the multiphase condensate.

(4) Run a genetic algorithm on one protein and systematically change the other protein to the target protein.

In our implementation of the genetic algorithm, we maintain a population of 20 sequences in each round, alongside a partner protein sequence that is not being evolved. To generate the initial population, we mutate the initial sequence by replacing, with 0.05 probability, each residue with a new one chosen from the 20 canonical amino acids with uniform probability. Each individual sequence **x** is assessed with a fitness function,1
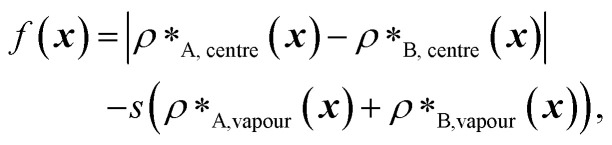
where 
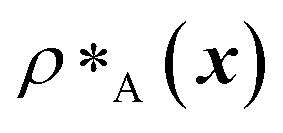
 and 
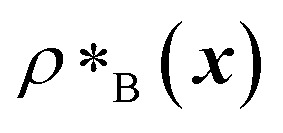
 are the average dimensionless number densities of the two different protein sequences A and B in the two-component mixtures. 
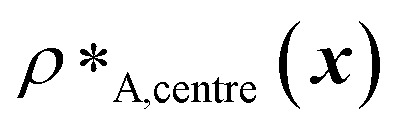
 and 
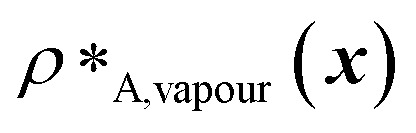
 denote the number density of protein A in the core of the multilayered condensate and in the dilute phase respectively, with analogous expressions for protein B [Fig. 1a]. Details of how these regions are determined are discussed in Section S1. The number densities are non-dimensionalised by dividing them with an appropriate unit, e.g. ρ* = *ρ*/nm^−3^, although the choice of unit is immaterial, since only the relative ordering in fitness is important, not the absolute numerical value. Finally, as discussed in the main text, the parameter *s* determines the trade-off between obtaining two stable liquid-like phases to give a stable multilayered condensate and the difference in compositions between the two phases. The value of *s* can be tuned as necessary depending on the specific system of interest; here, we have used *s* = 0, 0.5, 1 and 5.

The fitness function we have introduced is facile to compute and works well even in relatively small systems, which is especially useful in genetic-algorithm simulations where the fitness must be evaluated for many systems. Other order parameters have also been shown to identify compositional demixing within the condensed phase well, such as by quantifying the compositional asymmetry by considering the fraction of the two sequences in the two coexisting phases.^98^ However, in our simulations of relatively small system sizes, the structure and arrangement of the coexisting phases introduce extra complexity and it is difficult to determine accurately the density of the two components, especially in the outer layer of the condensate. Calculating the compositional asymmetry in this way would therefore likely not be straightforward enough to be used routinely in the genetic algorithm. Another approach uses intra- and inter-species pair correlation functions with demixing characterised by the intra-species pair correlation function dominating at small separations compared to the inter-species one.^99,100^ We compute the pair correlation functions for a range of our simulation outputs and show in Fig. S18† that this approach is consistent with the simpler fitness function we outlined above.

Once the fitness of each individual is determined, we use the tournament selection algorithm^52,114^ to select the fittest parents to cross over. We also apply a round of random mutations to explore previously unsampled regions of sequence space. Finally, we use a weak population replacement scheme to generate the population of sequences for the next round of the genetic-algorithm run. Our genetic-algorithm implementation is detailed in full in ref. 71.

The fitness of each individual in the population is computed when it is first encountered, *e.g.* following a mutation or crossover; however, when a systematic change is made to the partner sequence in coevolution runs, this too affects the phase behaviour and the fitness of all individuals in the population must therefore be recalculated. In our coevolution runs, we do this at 5-round intervals, at which we change ∼10% of the residues of this partner protein to the target protein sequence, with residues to be changed chosen randomly from those that have not yet been changed with uniform probability.

### 4.2 Simulation details

To simulate protein chains, we use the Mpipi residue-resolution sequence-specific coarse-grained model,^84^ combining (i) harmonic covalent bonds between residues, (ii) the Wang–Frenkel potential^115^ to account for non-bonded interactions between amino acids and (iii) Debye–Hückel electrostatic interactions.^116^ The Mpipi potential was shown to model LLPS of intrinsically disordered proteins well.^84^ For each amino-acid pair *ij*, the Mpipi model defines a Wang–Frenkel well-depth (*ε*_*ij*_), a characteristic length scale (*σ*_*ij*_) and values for *ν* and *μ* that determine the steepness of the potential well.

We use direct-coexistence simulations^117,118^ to model the vapour phase alongside the condensed phases in the same elongated tetragonal simulation box with explicit interfaces between phases. We use LAMMPS^119^ to run molecular-dynamics simulations with a typical time step of 10 fs and a coupling to a Langevin thermostat with a relaxation time of 10 ps. To estimate the densities of the dilute and condensed phases, we run each simulation for 40 ns for equilibration and an additional 20 ns to compute the densities. Each simulation takes around an hour on 76 CPU cores.

We use 96 chains of each protein for the evolution runs with generic sequences with a box size of 11.4 nm × 11.4 nm × 56.9 nm. For coevolution runs, we use 45 chains of the protein that is changed to A1 LCD, and either 90 chains of 50 residues or 45 chains of 100 residues of the other protein, in a box of size 10.9 nm × 10.9 nm × 54.7 nm. Although finite-size effects were examined in ref. 84 with similar-sized systems, we simulate several systems obtained in coevolution runs with A1 LCD concentrated in the inner layer at larger system sizes to check that the density profiles are consistent. We do this separately for a system where the final evolved system is highly multiphasic and the two condensed phases are essentially pure [Fig. S21†], and a system where there is still a considerable degree of mixing of the two proteins in the condensed phases [Fig. S22†]. The results from increasing system sizes appear to suggest that the outer phase is not merely wetting the surface of the inner phase but forms a layer that scales with the system size and is likely to be a genuine immiscible phase. Multiphasic biomolecular condensates reported in the experimental literature are often relatively small, and so may be stabilised by interfacial considerations^120^ rather than by forming truly immiscible bulk phases. If these phases are true thermodynamic phases, although they may of course have a preferred ordering in direct-coexistence simulations because of interfacial free-energy considerations, each of the three phases in question should be able to coexist independently with any one of the others under the same thermodynamic conditions. We test whether this holds for representative systems with different underlying multiphasic behaviour and confirm that each of the condensed phases coexists with the vapour-like phase under the same conditions as in the multiphasic regime [Fig. S23†]. For the systems investigated, as previously implied by finite-size scaling, these therefore appear to be genuine thermodynamic phases. Finally, we also perform a sanity check by verifying that if we double the system size, the ordering of fitness values is the same [Fig. S24†].

To ensure that our predictions are robust, we have confirmed that the final predicted sequences obtained from genetic-algorithm runs with the Mpipi potential exhibit similar multiphasic behaviour when simulated with another coarse-grained potential, namely Model 2 of ref. 87.

### 4.3 Interfacial free-energy densities

We compute interfacial free-energy densities for the interface between the vapour-like phase and the pure condensed phase for the final evolved maximum-fitness sequences in coevolution runs with A1 LCD for both cases, *i.e.* where the coevolved protein is the inner or the outer protein. To do this, we use the Kirkwood–Buff expression^121^ to relate the interfacial free-energy density *γ* to the normal and tangential components of the pressure tensor, and the mean value theorem to simplify the result^122^ for planar interfaces into *γ* = (*L*_*z*_/2)(*P*_norm_ − *P*_tang_), where *γ* is the interfacial free-energy density, *L*_*z*_ is the length of the simulation box along which the interface occurs, *P*_norm_ = *P*_*zz*_ is normal to the interface and *P*_tang_ = *P*_*xx*_ = *P*_*yy*_ is the tangential pressure, and the division by 2 accounts for the fact that there are two interfaces in our simulation set-up.^120^ Although the pressure tensor has many possible definitions, from virial to mechanical expressions, the interfacial-free energy density is independent of this arbitrary choice;^123^ we use the atomic virial pressure tensor in our calculation, which gives the same results as the molecular virial.^124^

We compute only the interfacial free-energy density for the interface between the dense and dilute phases (*i.e.* a surface tension using our coarse-grained model), since as long as a multilayered condensate forms, the interface between the two condensed phases of different compositions is always present and therefore does not affect the thermodynamics. We assume for simplicity that the resulting phases are pure and, in this back-of-the-envelope calculation, we do not consider the possible dependence of *γ* on the interface width or the curvature of the droplet. We determine the interfacial free-energy density for each system at several temperatures. The resulting data are well fitted with a linear function [Fig. S12a†]; since (∂*γ*/∂*T*) = −*S*_int_, the interfacial entropy,^106^ this approach allows us to extract the interfacial energy and interfacial entropy for each component, as discussed in the main text. We test for finite-size effects in the interfacial free-energy density as a function of both the surface area and the bulk depth and see that the values are the same within error bars across different system sizes [Fig. S12b†].

## Data availability

All relevant data are within the manuscript, its ESI† files and the Figshare data repository at https://doi.org/10.6084/m9.figshare.21926154.

## Code availability

LAMMPS input scripts are available in the Figshare data repository at https://doi.org/10.6084/m9.figshare.21926154.

## Author contributions

PYC, JAJ, RC-G and AR designed the research. PYC performed the research. PYC, JAJ, RC-G and AR analysed the results and wrote the paper.

## Conflicts of interest

The authors declare no competing interests.

## Supplementary Material

SC-014-D2SC05873A-s001
