# Supplementary material for: Thermodynamic origins of two-component multiphase condensates of proteins
Source: Chem Sci. 2023 Jan 25;14(7):1820–36. doi: 10.1039/d2sc05873a (PMC9931050; doi:10.1039/d2sc05873a)
Supplement: SC-014-D2SC05873A-s001 [file SC-014-D2SC05873A-s001.pdf]

Electronic Supplementary Material (ESI) for Chemical Science.
This journal is © The Royal Society of Chemistry 2023

Supporting Information:
Thermodynamic origins of two-component multiphase condensates of proteins

Pin Yu Chew,[1] Jerelle A. Joseph,[1,2,3] Rosana Collepardo-Guevara,[1,2,3,a)] and Aleks Reinhardt[1,b)]

[1)] *Yusuf Hamied Department of Chemistry, University of Cambridge, Cambridge, CB2 1EW, United Kingdom*

[2)] *Department of Physics, University of Cambridge, Cambridge, CB3 0HE, United Kingdom*

[3)] *Department of Genetics, University of Cambridge, Cambridge, CB2 3EH, United Kingdom*

(Dated: 2022-10-24)

S1 DETERMINING THE CENTRE OF THE DENSITY PROFILE

To calculate the density profiles, the simulation box is divided into 150 bins along the elongated axis, and the average density of each species is calculated within each bin. The 'centre' region of the initial reference system is taken as the region between where the density profiles of the two protein species intersect. The 'vapour' region is then defined as the 50 bins in total where the first and last bin are equidistant from the middle of the 'centre' region. Once these regions are quantified for the initial reference system, we fix the centre of mass of the condensate in our simulations and keep these regions constant throughout the entire genetic algorithm run.

S2 EFFECT OF WEIGHTING PARAMETER s IN THE FITNESS FUNCTION

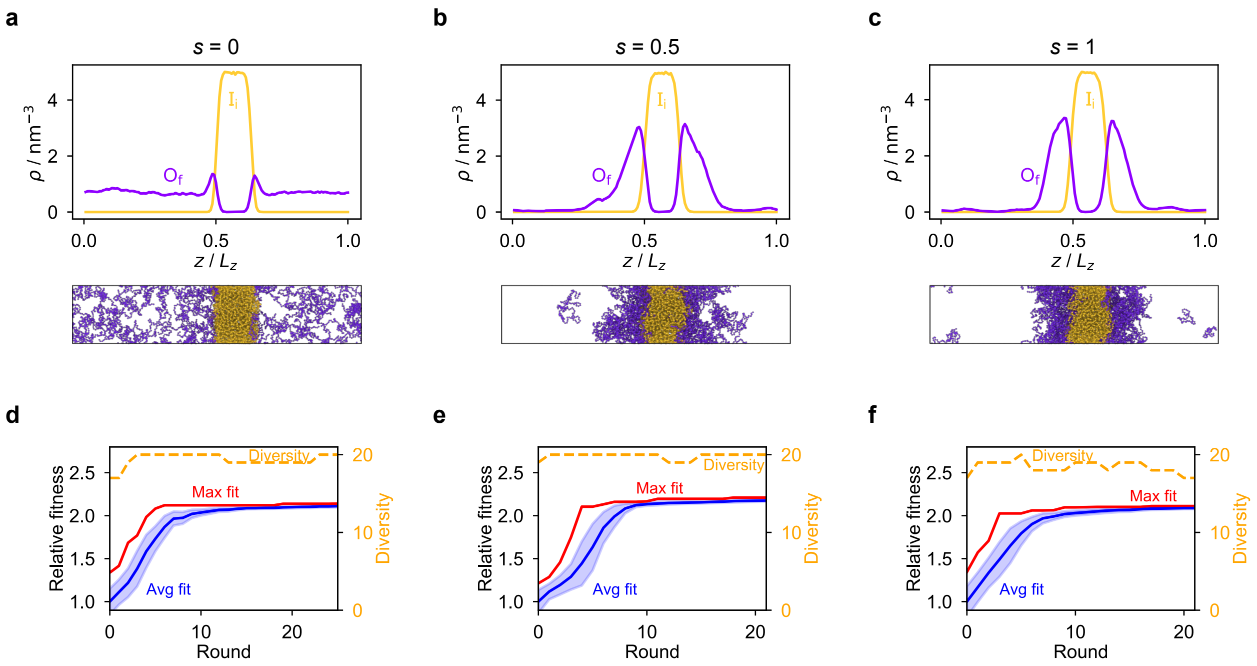

Figure S1. **Effect of weighting parameter s in the fitness function.** Density profiles of the final evolved systems with maximum fitness using (a) $s = 0$, (b) $s = 0.5$ and (c) $s = 1$ in the fitness function of the genetic algorithm for the evolution run where the outer sequence is evolved and the inner sequence is kept unchanged. In this case, the quality of the final result is dependent on the value of s. When $s = 0$, the fittest individual corresponds to a sequence which results in a system with a dense phase of one component and a dilute phase of the other, instead of two liquid-like phases with different compositions in a multilayered arrangement. To obtain the latter, $s > 0$ was needed in the evolution run. (d–f) Genetic algorithm progressions for the three cases with different values of s. Shaded area for the average fitness corresponds to the standard deviation across all the sequences present in the population at each round.

S3 COEVOLUTION OF A1 LCD WITH OTHER REFERENCE SYSTEMS

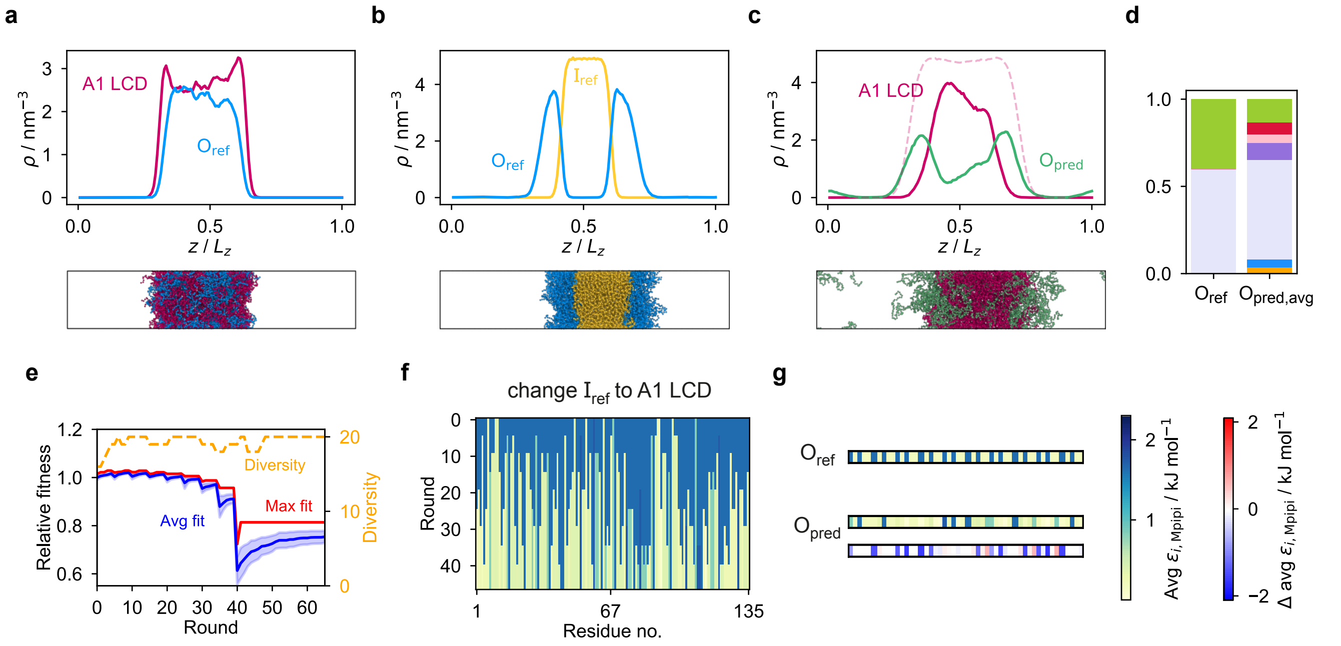

Figure S2. **Coevolution of A1 LCD with a shorter partner protein.** Coevolution run with A1 LCD designed to be the inner sequence with a shorter partner sequence of 50 residues as the outer sequence. (a) System with low multiphasicity obtained when the systematic change to A1 LCD is made directly all at once from (b) the initial reference system with high multiphasicity. $\mathrm{I_{ref}} = \mathrm{F_{135}}$ and $\mathrm{O_{ref}} = (\mathrm{FAFAA})_{10}$. (c) Final evolved system with maximum fitness obtained from the coevolution approach. In this case, using a shorter partner sequence on the outside gives a result with a lower degree of multiphasicity compared to when a partner sequence of twice the length is used. (d) Comparison of the initial and final compositions of the evolved sequence. Colours used to represent the different types of amino acids are the same as in Fig. 3 of the main text. (e) Genetic algorithm progression of this coevolution run. Shaded area for the average fitness corresponds to the standard deviation across all the sequences present in the population at each round. With a shorter partner sequence, the maximum fitness does not improve beyond round 40 after all the systematic changes has been made. (f) Illustration of how the systematic change from $\mathrm{I_{ref}}$ to A1 LCD is made. (g) Comparison of the final evolved sequence with maximum fitness, $\mathrm{O_{pred}}$, with the initial reference sequence $\mathrm{O_{ref}}$. To illustrate the sequences in (f–g), we plot for each residue i along the sequence the absolute value and the change in the value of $\varepsilon_{i,\mathrm{Mpipi}}$ compared to the initial reference sequence.

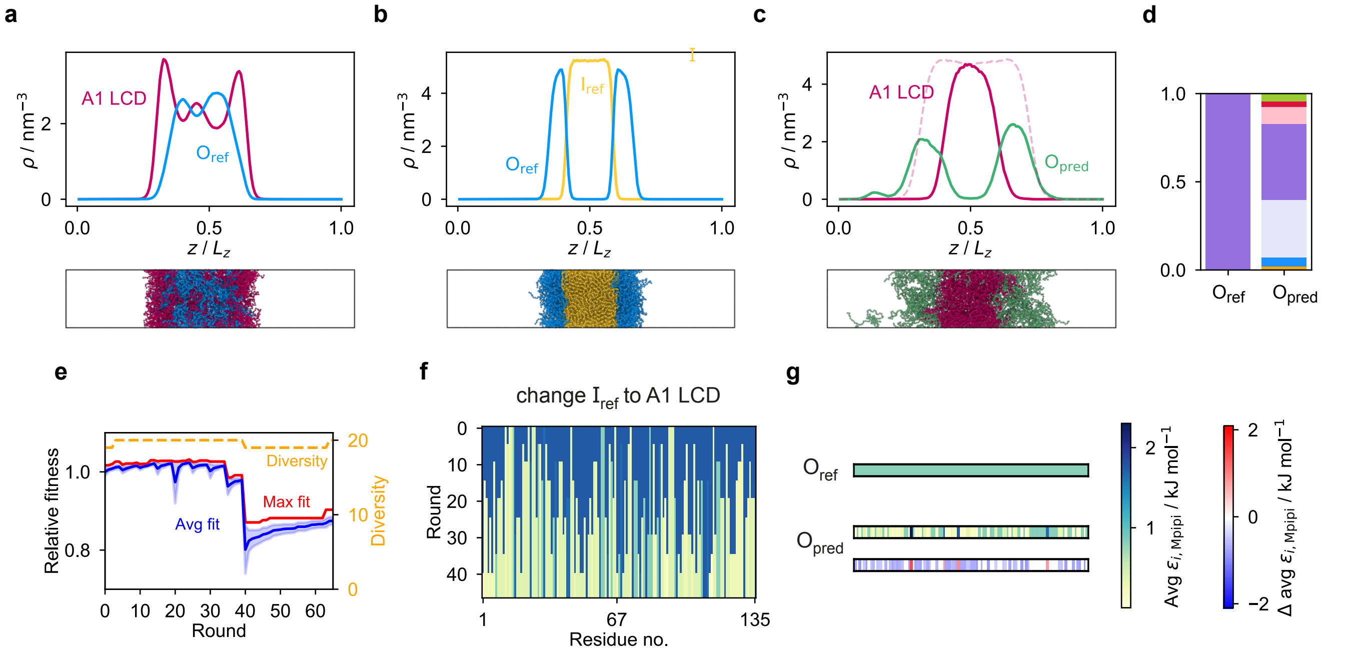

Figure S3. **Coevolution of A1 LCD from different reference proteins.** The analogue of Fig. S2 starting from a different initial reference system, namely $\mathrm{I_{ref}} = \mathrm{Y_{135}}$ and $\mathrm{O_{ref}} = \mathrm{N_{100}}$.

S4 SYSTEMATIC CHANGES DURING COEVOLUTION RUNS

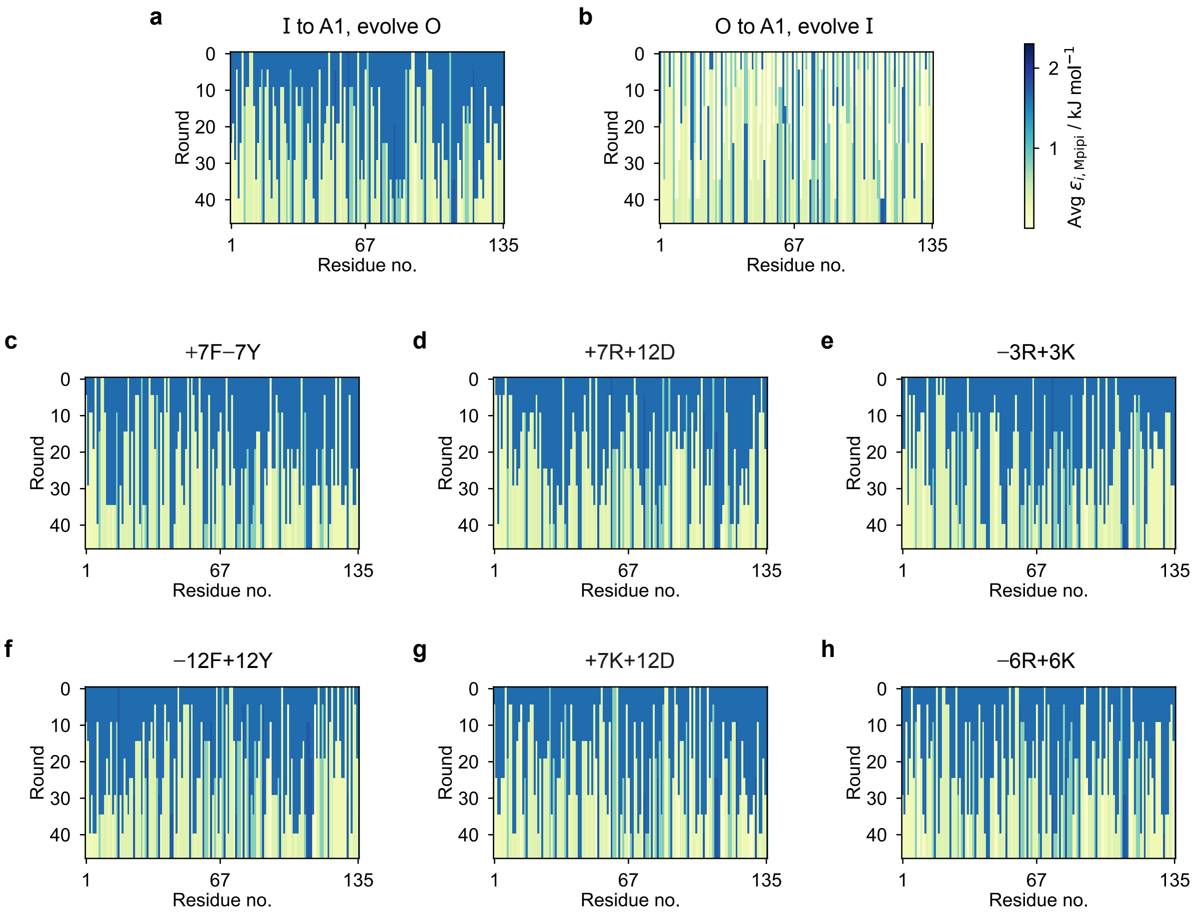

Figure S4. **Manner in which the systematic changes are made in the coevolution runs with A1 LCD and its variants.** We illustrate the sequence of the protein that is systematically changed to the A1 variant in each round by plotting the value of $\varepsilon_{i,\mathrm{Mpipi}}$ for each residue i along the sequence.

S5 GENETIC-ALGORITHM PROGRESSIONS FOR COEVOLUTION RUNS

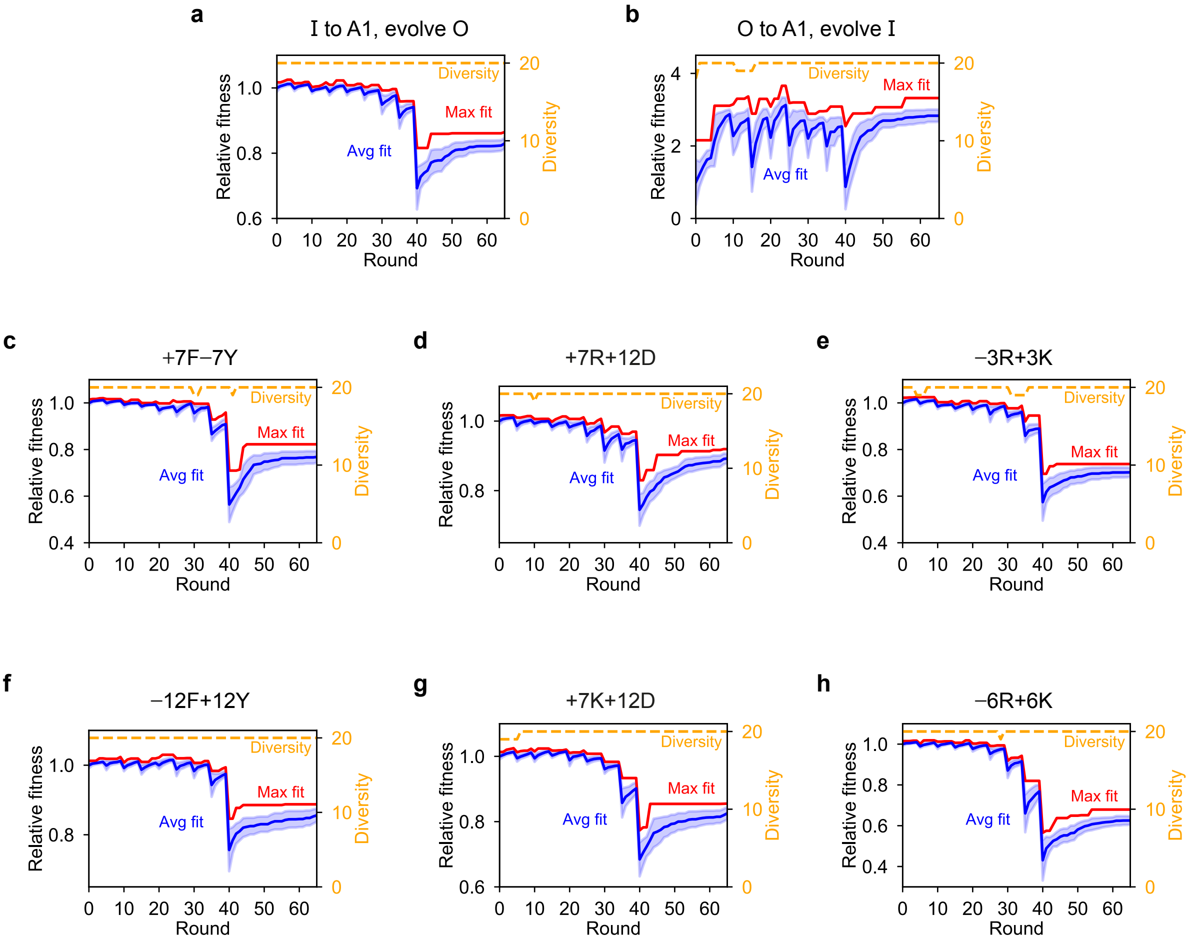

Figure S5. **Genetic-algorithm progressions for coevolution runs with A1 LCD and its variants.** Shaded areas for the average fitness correspond to the standard deviation across all the sequences present in the population at each round.

S6 COEVOLVING A1 LCD AT THE CENTRE OR ON THE OUTSIDE

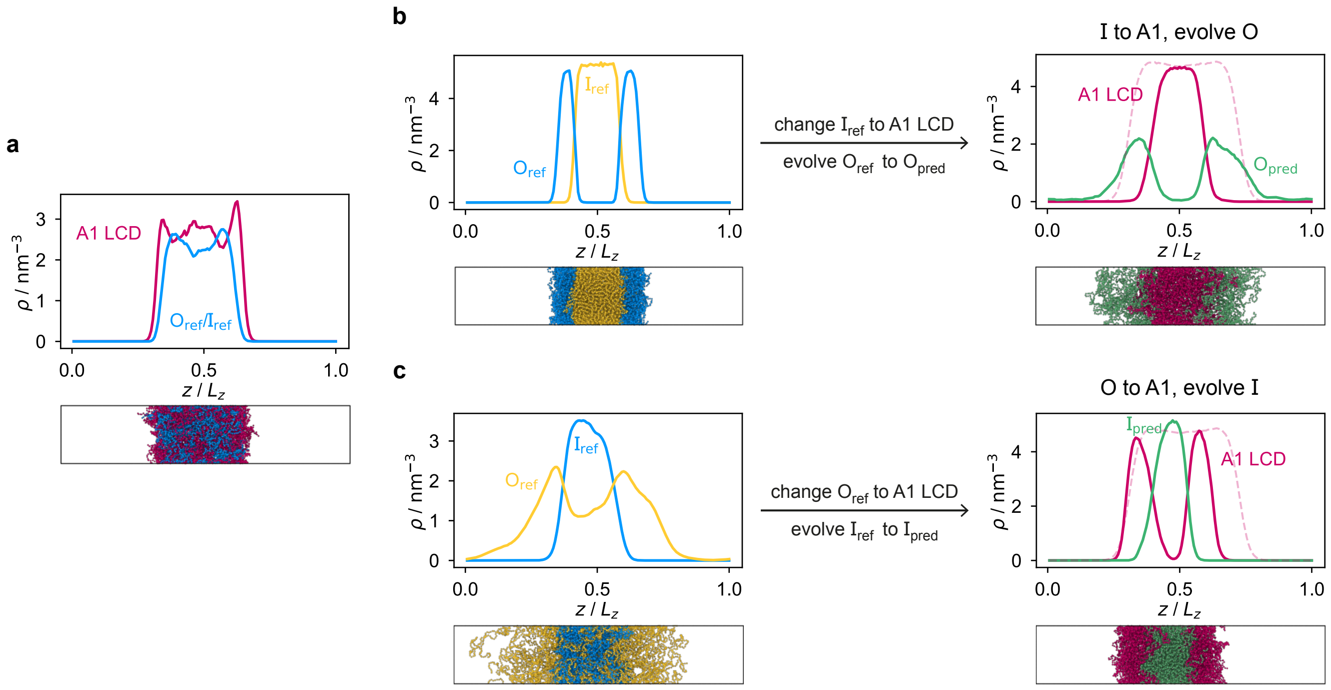

Figure S6. **Designing multilayered condensates with A1 LCD concentrated at the centre or in the outer layer.** (a) System with low multiphasicity obtained when the systematic change to A1 LCD is made directly all at once from the two initial reference systems with high multiphasicity. O_{ref}/I_{ref} = $(FAFAA)_{20}$. (b) Density profiles of the initial reference system and final evolved system for the case where A1 LCD is designed to be the inner sequence. I_{ref} = F_{135} is systematically changed to A1 LCD and O_{ref} = $(FAFAA)_{20}$ is evolved using the genetic algorithm. (c) Density profiles of the initial reference system and final evolved system for the case where A1 LCD is designed to be the outer sequence. O_{ref} = $(FIQII)_{27}$ is systematically changed to A1 LCD and I_{ref} = $(FAFAA)_{20}$ is evolved. The pink dashed lines in the final evolved systems of both cases is the density profile of a single-component system of A1 LCD equilibrated at the same temperature. Note that O_{ref} in panel (b) is the same as I_{ref} in panel (c).

S7 CHANGES IN INTERACTION ENERGIES DURING GENETIC-ALGORITHM RUNS

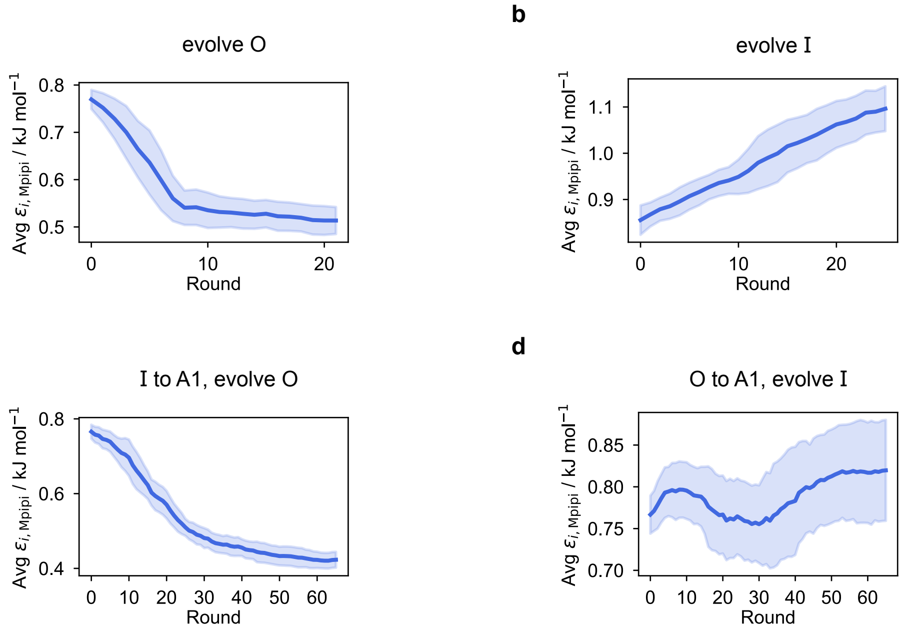

Figure S7. **Changes in the average value of $\varepsilon_{i,\mathrm{Mpipi}}$.** These are shown for the evolution runs where (a) the outer sequence is evolved and the inner sequence is kept unchanged and (b) the inner sequence is evolved and the outer sequence is kept unchanged, and for the coevolution runs where A1 LCD is designed to be (c) the inner sequence or (d) the outer sequence in the final multilayered system. The value of $\varepsilon_{i,\mathrm{Mpipi}}$ plotted is averaged over all the residues of the evolved sequences in the entire populations in three independent runs for each case. Shaded areas correspond to the standard deviation.

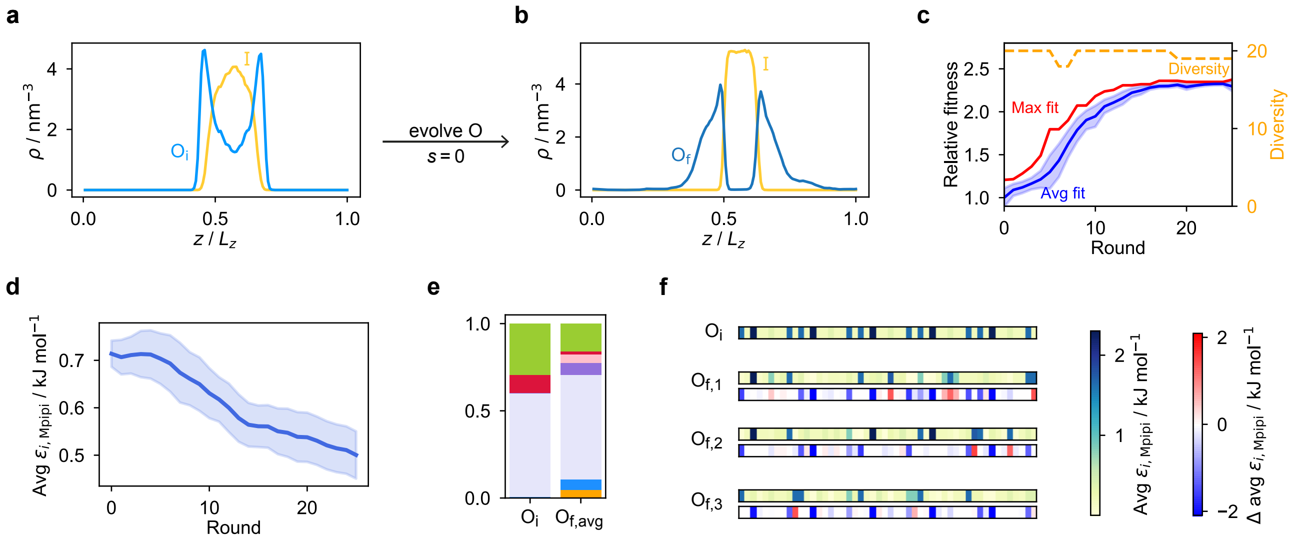

Figure S8. **Evolving the outer sequence while keeping the inner sequence unchanged from a different initial system.** (a) Density profile of initial system with low multiphasicity. I = F_{50} and O_i = $(FAWAARAAFA)_5$. (b) Density profile of final evolved system with maximum fitness displaying high multiphasicity. (c) Genetic algorithm progression of this evolution run. Shaded area for the average fitness corresponds to the standard deviation across all the sequences present in the population at each round. (d) Change in the average value of $\varepsilon_{i,\mathrm{Mpipi}}$ over the evolution run. The value of $\varepsilon_{i,\mathrm{Mpipi}}$ plotted is averaged over all the residues of the evolved sequences in the entire populations in three independent runs and shaded areas correspond to the standard deviation. (e) Comparison of the initial and final compositions of the evolved sequence. Colours used to represent the different types of amino acids are the same as in Fig. 3 of the main text. (f) Comparison of the final evolved sequences with maximum fitness in three independent runs, O_f, with the initial reference sequence O_i. To illustrate the sequences, we plot for each residue i along the sequence the absolute value and the change in the value of $\varepsilon_{i,\mathrm{Mpipi}}$ compared to the initial reference sequence. Overall, the results obtained from a different initial system are consistent with those presented in the main text where the outer sequence was also evolved.

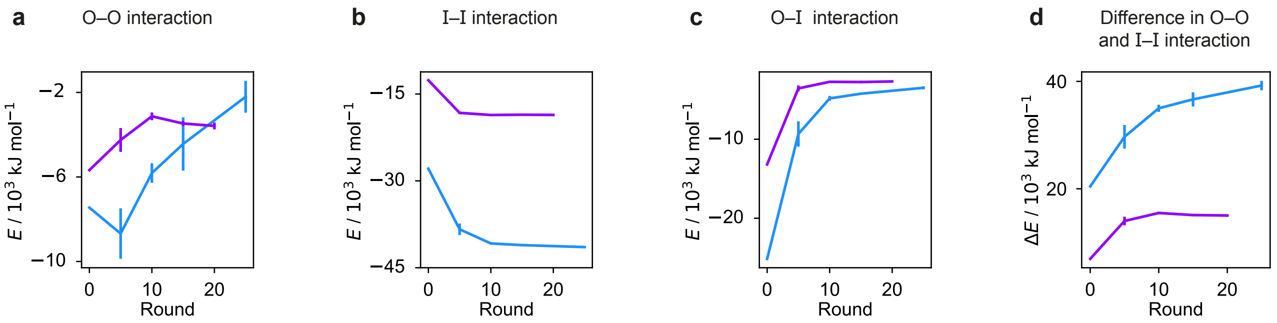

Figure S9. **Interaction-energy trends appear to be robust to initial sequence choice.** The purple curve is reproduced from Fig. 4. The changes in interaction energies for an evolution run where the outer sequence is evolved and the inner sequence is kept unchanged starting from a different initial system [Fig. S8], shown in blue, show similar trends to the curves in purple.

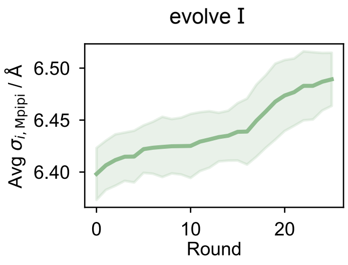

Figure S10. **Change in the average value of $\sigma_{i,\mathrm{Mpipi}}$.** This is shown for the evolution run in which the inner sequence is evolved and the outer sequence is kept unchanged [Fig. 1(b–d) of the main text]. The value of $\sigma_{i,\mathrm{Mpipi}}$ plotted is averaged over all the residues of the evolved sequences in the entire populations in three independent runs for each case. Shaded areas correspond to the standard deviation. By contrast, there is no clear trend in the behaviour of $\sigma_{i,\mathrm{Mpipi}}$ in the evolution run where the outer sequence is evolved.

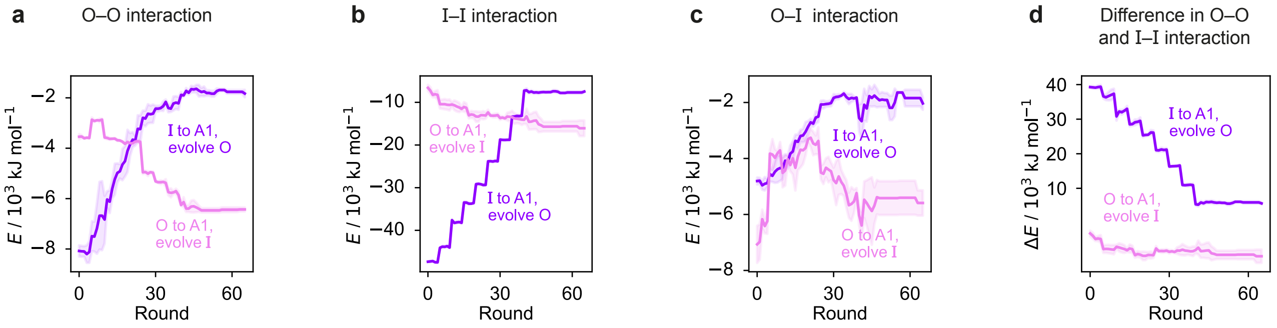

Figure S11. **Change in the interaction energies during genetic-algorithm progression for coevolution runs.** We show these changes for intermolecular self-interactions between proteins enriched in (a) the outer and (b) the inner layer, (c) cross-interactions between the two proteins and (d) the difference in the O–O and I–I interaction energies for the system with the fittest individual in the coevolution runs where one sequence is changed to A1 LCD and the other is coevolved. Shaded areas correspond to the standard deviation across three independent runs. Although the difference in O–O and I–I interactions decreases overall, this is because of the systematic changes made to the sequence. The trends for the genetic-algorithm rounds in between those at which systematic changes occur are largely consistent with the results shown in Fig. 4.

S8 INTERFACIAL FREE-ENERGY DENSITIES

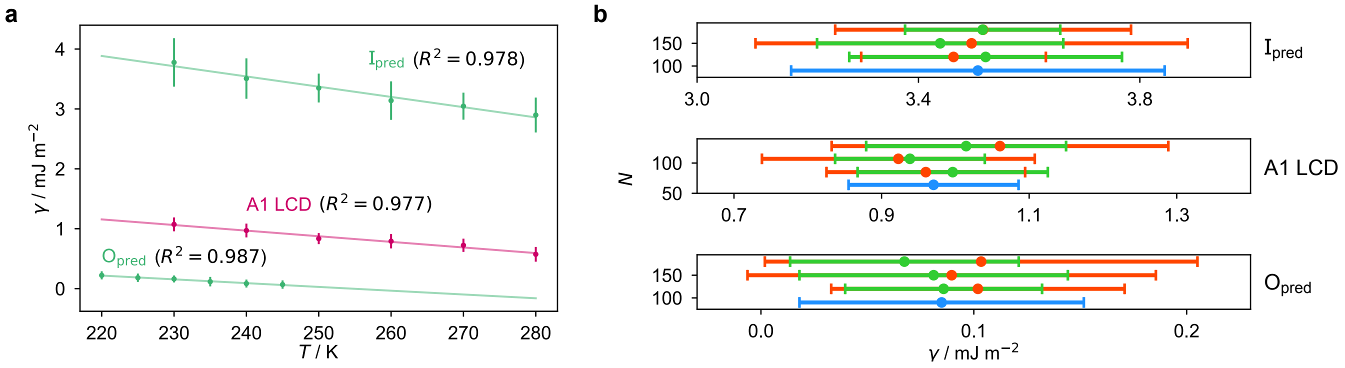

Figure S12. **Interfacial free-energy densities.** (a) Interfacial free-energy densities as a function of temperature. Error bars correspond to the standard deviation of the interfacial free-energy density computed in several independent sections of the simulation. We use a linear fit to the data points to extract the interfacial entropy and energy for each sequence, as discussed in the main text. (b) Finite-size scaling of the interfacial free-energy densities. We have computed the interfacial free-energy density of the three sequences at 240 K with larger system sizes, varying both the bulk depth (red) and surface area (green). The interfacial free-energy density of the original system size, plotted in (a), is shown in blue. For all three sequences, there is no difference within error bars in the value of the interfacial free-energy density across the different system sizes tested.

S9 COMPOSITION AND PATTERNING IN COEVOLUTION RUNS

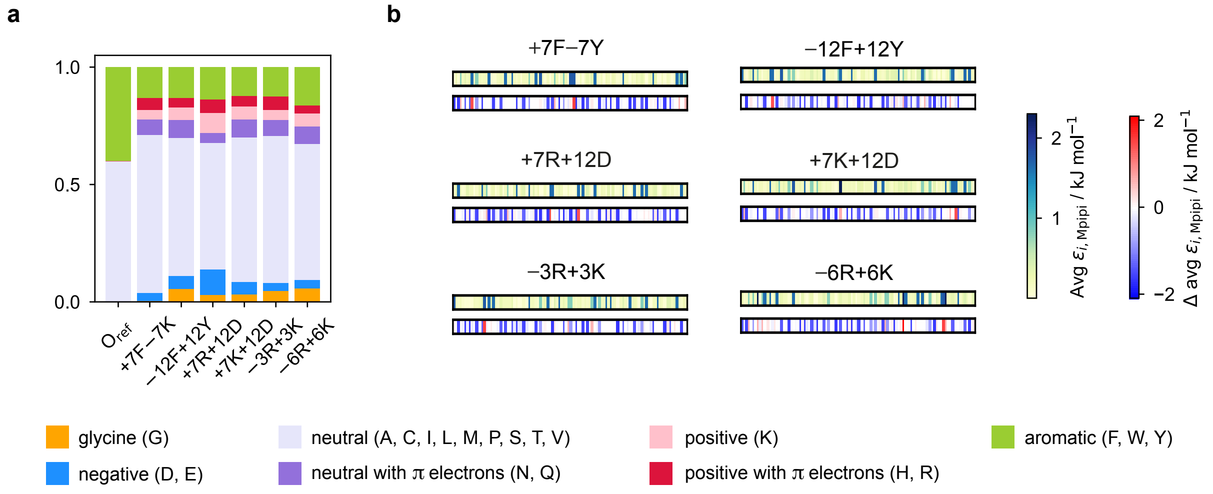

Figure S13. **Composition and patterning in coevolution runs.** (a) The amino-acid composition and (b) patterning of the final evolved sequences with maximum fitness in the coevolution runs with 6 different variants of A1 LCD designed to be in the centre of the final multilayered condensate. In (b) we plot the absolute value and the change in the value of $\varepsilon_{i,\mathrm{Mpipi}}$ compared to the initial reference sequence for each residue i along the final evolved sequence. In all cases we observe a decrease in the proportion of aromatic residues, and the final sequences contain less strongly interacting residues overall.

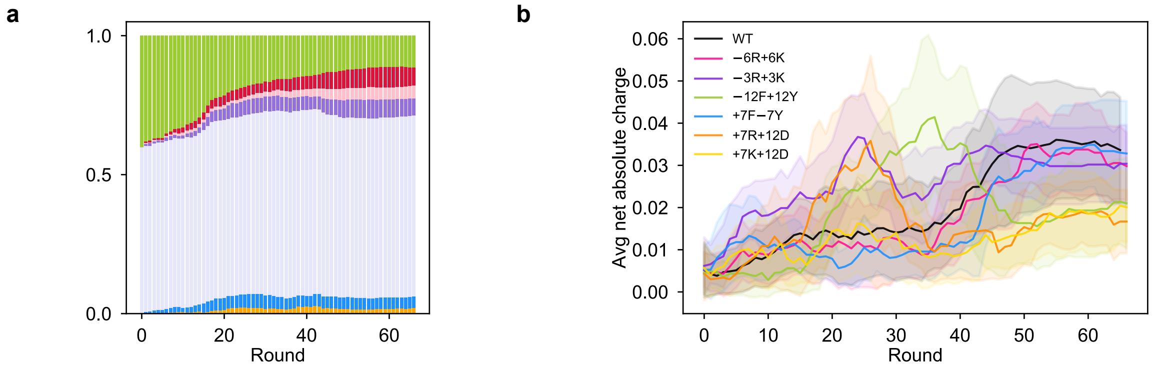

Figure S14. **Changes in composition and net charge throughout the coevolution runs.** (a) The change in amino-acid composition of the evolved sequence throughout the coevolution run where A1 LCD is designed to be the inner sequence. (b) The change in average net absolute charge of the evolved sequences in the coevolution runs with 7 variants of A1 LCD (including the WT) designed to be in the centre of the final multilayered condensate. The absolute net charge is averaged over all the evolved sequences in the population at each round. Shaded areas correspond to the standard deviation.

S10 EFFECT OF STICKER AND CHARGE PATTERNING ON MULTIPHASICITY

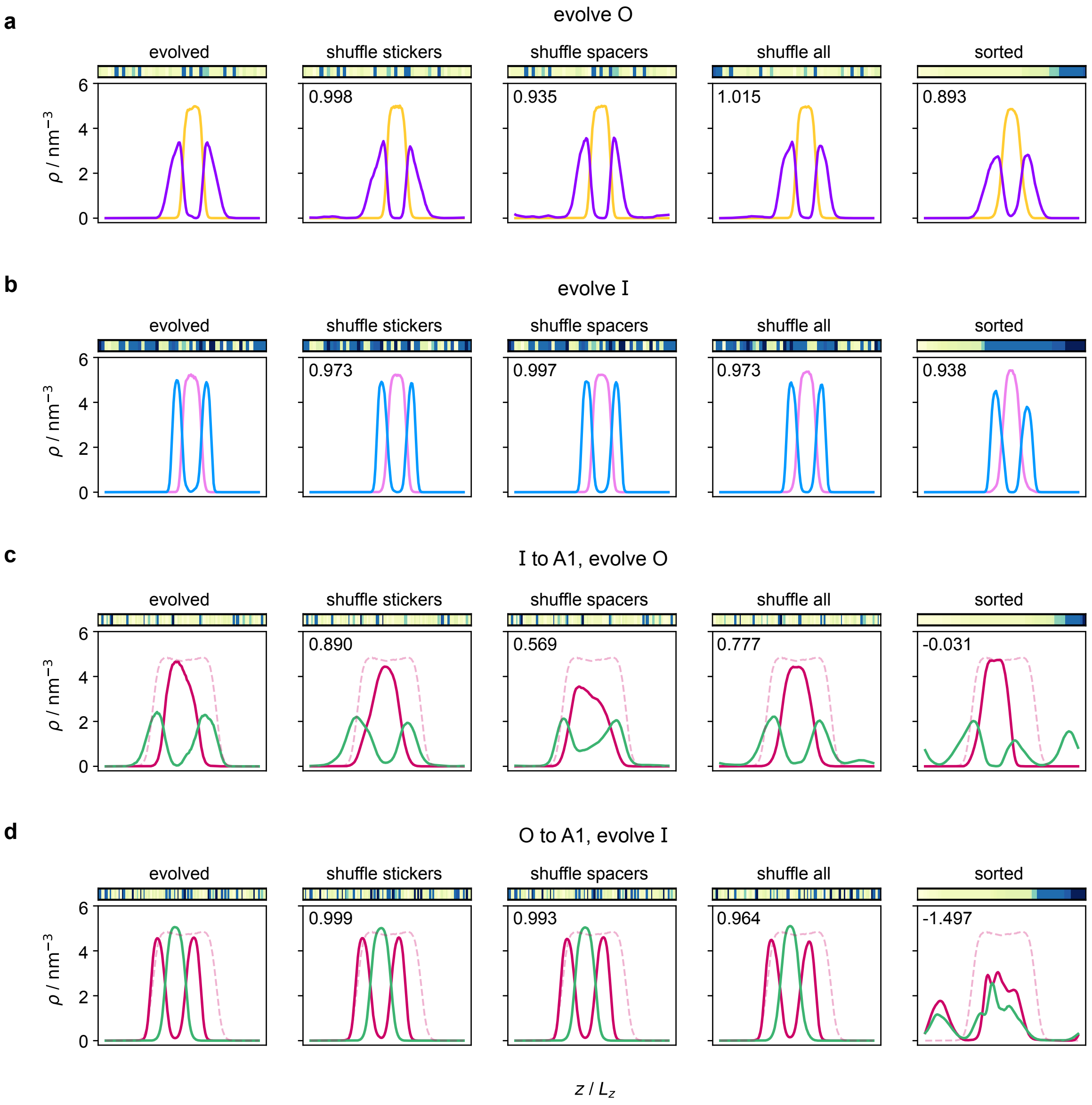

Figure S15. **Sequence patterning only sometimes affects multiphasicity.** Density profile of the final evolved system with maximum fitness in (a–b) evolution and (c–d) coevolution runs with A1 as the partner sequence, with inner or outer proteins evolved as indicated. The first column corresponds to the final evolved system with maximum fitness in each case, reproduced from the main text [(a) = Fig. 1(c); (b) = Fig. 1(d); (c) = Fig. 2(b); and (d) = Fig. 2(c)]. The remaining columns correspond to different shuffles of the same sequence, as indicated, using the same notation and colours as in the main text. Just above each density profile, we show a map of $\varepsilon_{i,\mathrm{Mpipi}}$ values for the sequence in question, as in Fig. 5. In the top left-hand corner of each density plot, we give the fitness value relative to the evolved sequence of the left-most column. In the case of panels (a) and (b), even if amino acids are completely sorted by their interaction strength in the sequence, the multiphasicity is not appreciably reduced, suggesting that patterning is not crucial in these cases. By contrast, even just shuffling the spacers in panel (c) leads to a considerable reduction in fitness, whilst sorting the amino acids results in very different phase behaviour in panels (c) and (d), indicating that patterning is important in these cases.

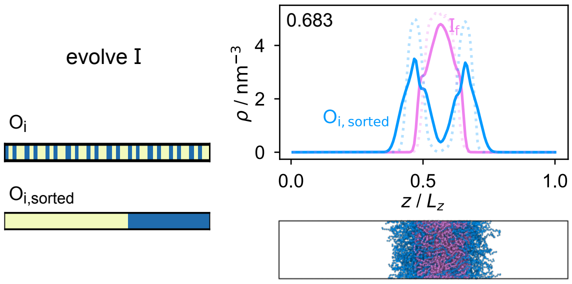

Figure S16. **Density profile for sorted partner sequence.** Density profile and simulation snapshot of the final evolved system with maximum fitness in the evolution run where the inner sequence is evolved, but with the originally fixed partner sequence $\mathrm{O_i} = (\mathrm{FAFAA})_{10}$ sorted such that residues of the same type are all clustered together. The density profile of the original evolved system is shown by the dotted lines. The number in the top left corner of the density plot is the fitness value relative to the original system with the unsorted partner sequence.

As discussed in the main text, compositional demixing has been found to be favoured in two-component systems with a high charge pattern mismatch.[1,2] Charge patterning can be characterised by the blockiness parameter κ[3] or by the sequence charge decoration (SCD), defined by[4]

$$Q_{\mathrm{SCD}} = \frac{1}{N} \sum_{i=1}^{N} \sum_{j=i+1}^{N} z_i z_j \sqrt{j-i}, \tag{S1}$$

where z_i is the charge number of the residue at position i along the chain and N is the number of amino-acid residues in the protein sequence. To investigate the effect of charge pattern mismatch on multiphasicity, we show in Fig. S17 the difference in Q_{SCD} between the two protein sequences for the systems in Fig. S15(c) and additional systems with residues of the same charge grouped together. Since the phase behaviour of the systems considered here is not principally charge-driven, it is perhaps not surprising that we find a low correlation between the charge pattern mismatch and the fitness.

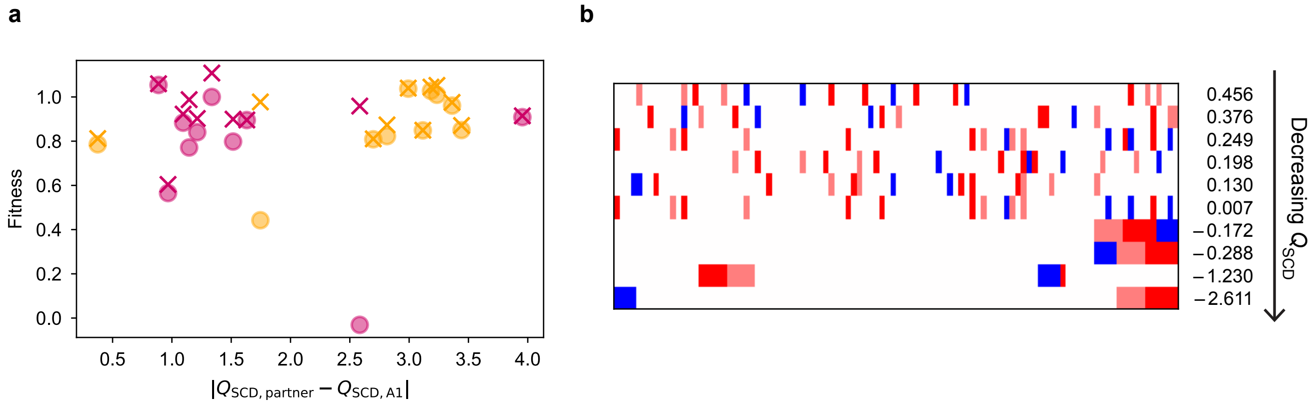

Figure S17. **Correlation between charge pattern mismatch and fitness.** (a) Scatter plot of fitness values against the difference in charge patterning of the two sequences in the system, quantified using the difference in SCD values, for the systems in Fig. S15(c), as well as additional systems with the charged residues blocked together. The points for systems of the partner sequences with the WT A1 LCD ($Q_{\mathrm{SCD}} = 1.344$) are plotted in pink, while the points for the systems with A1 LCD sorted with residues in order increasing charge ($Q_{\mathrm{SCD}} = -2.986$) are in orange. Points with the fitness function calculated with $s = 5$ and $s = 0$ are plotted with circles and crosses respectively. Fitness values are normalised by the fitness of the system with the original predicted partner and A1 LCD [Fig. 2(b)]. The Pearson correlation and Spearman's rank correlation coefficients are 0.125 and 0.329 for $s = 5$ (circles), and 0.175 and 0.0662 for $s = 0$ (crosses), respectively. (b) Schematic of the partner sequences considered in (a) represented as a map of the charge of each residue, with their SCD values listed on the right of each sequence. Residues with positive and negative charges are shown in red and blue respectively (histidine, which has half the positive charge of lysine and arginine in the Mpipi model, is shown in pink).

One thing to note about the patterning parameters Q_{SCD} and κ, as well as the sticker pattering parameter Ω_{aro}[5] equivalent of κ, is that studies investigating patterning based on these parameters usually look at sequences with the same overall composition, and comparing the value of these patterning parameters is less meaningful for sequences of different compositions.[6] Consequently, we have here compared the Q_{SCD} values for shuffled sequences of the same system with the same overall composition. However, since sequences change over the course of a genetic-algorithm run, it would be rather less straightforward to compare charge patterning.

S11 IDENTIFYING COMPOSITIONAL DEMIXING USING PAIR CORRELATION FUNCTIONS

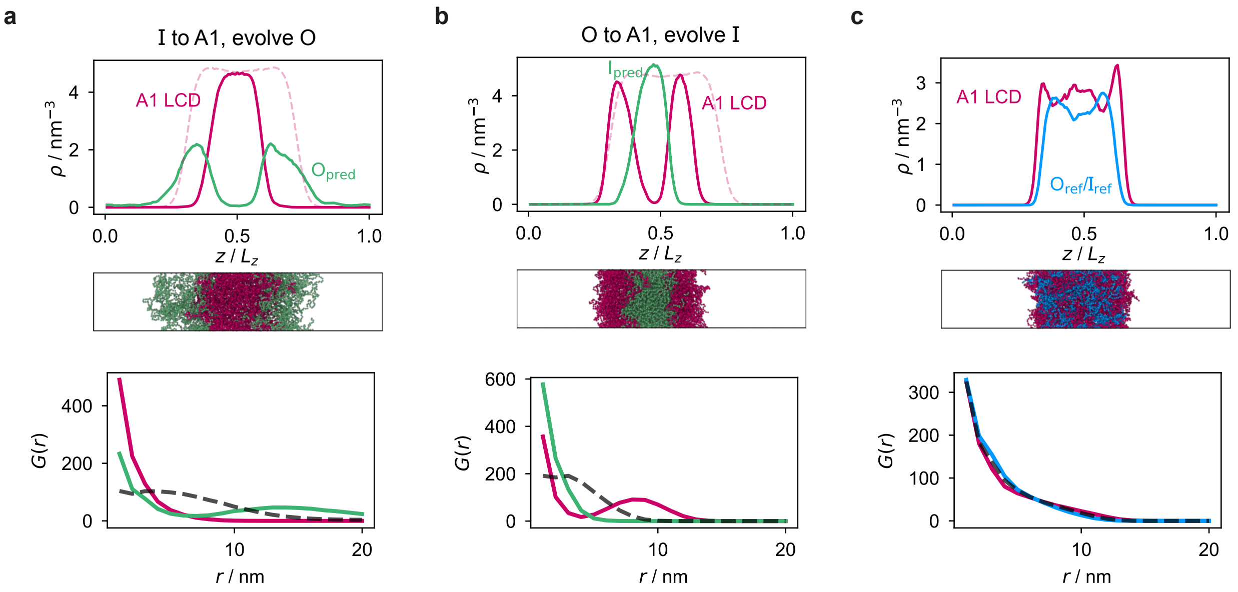

Figure S18. **Multiphasicity can be identified using pair correlation functions.** Pair correlation functions $G(r)$ of (a, b) the final systems obtained in the coevolution runs with A1 LCD with high multiphasicity [(a) = Fig. 2(b); (b) = Fig. 2(c)] and (c) a well-mixed condensate with low multiphasicity [Fig. S6(a)]. In each case, the intra-species pair correlation function is shown by solid lines in the same colour as the corresponding protein in the density profiles and simulation snapshots, while the inter-species pair correlation function is shown by the black dashed line. For the systems with high multiphasicity, the intra-species $G(r)$ dominates at small r over the inter-species $G(r)$, whereas they are comparable for the well-mixed system. The pair correlation function $G(r)$ is calculated as a histogram of the intermolecular distances, and the intermolecular separation r is taken to be the shortest of the possible interbead separations, accounting for periodic boundary conditions.

S12 TESTING THE COEVOLUTION APPROACH WITH GENERIC SEQUENCES

As a proof of principle, we first tested the coevolution approach on simple two-component systems with generic sequences before applying it to biologically relevant proteins like A1 LCD as detailed in the main text.

For these coevolution runs, the initial reference system we used is a mixture of F_{50} and $(FAFAA)_{10}$, which form a multilayered condensate with two compositionally distinct liquid-like phases of high purity: F_{50} concentrates in the centre while $(FAFAA)_{10}$ remains in the outer layer. We selected two target sequences into which either one of F_{50} or $(FAFAA)_{10}$ is systematically changed whilst the other is simultaneously evolved using the genetic algorithm. These target sequences are selected such that making the systematic change directly in one go would result in complete mixing to give one homogeneous liquid-like phase. The systematic change from the initial reference sequence to the target sequence is made by changing 10 % of the residues every 5 rounds [Fig. S19(e) and Fig. S20(e)].

In Fig. S19(b–c) and Fig. S20(b–c), we show the density profiles and snapshots at the start and end of the coevolution runs; the final systems clearly exhibit two liquid-like phases of different composition, demonstrating that the coevolution approach is able to predict a partner sequence that can form a multilayered condensate with another sequence of interest. The approach is successful both when the target sequence is designed to be concentrated at the centre or on the outside of the multilayered condensate. The genetic algorithm progressions in the two cases are shown in Fig. S19(d) and Fig. S20(d). The rounds where a drop in the average and maximum fitness is observed broadly corresponds to rounds where systematic changes are made.

The final evolved sequence with maximum fitness in the two different evolution cases in terms of the approximate interaction strengths of the residues are shown in Fig. S19(f) and Fig. S20(f).

The behaviour of these generic sequences across the genetic-algorithm progression both in terms of fitness and in terms of the types of interaction that favour multiphasicity is very similar to the case of coevolution with A1 LCD discussed in the main text, even though the generic sequences we have used are considerably shorter than A1 LCD, suggesting that these results are largely independent of the specifics of the amino-acid sequences in question.

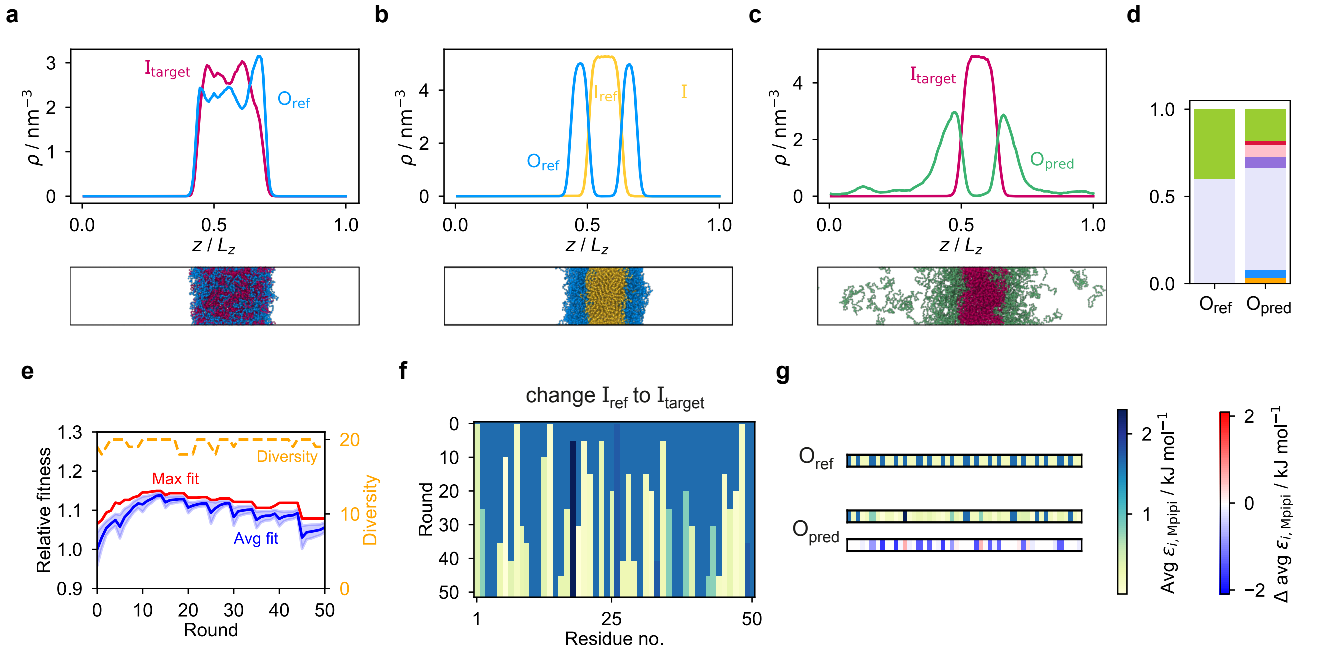

Figure S19. **Coevolution with generic sequences (I).** Coevolution run with generic sequences where the inner sequence is systematically changed and the outer sequence is evolved. I_{ref} = F_{50}, O_{ref} = $(FAFAA)_{10}$ and I_{target} = mutated $(FAFAA)_{10}$, with a mutation probability of 0.60. (a) System with low multiphasicity obtained when the systematic change is made directly all at once from (b) the initial reference system with high multiphasicity. (c) Final evolved system with maximum fitness. (d) Comparison of the initial and final compositions of the evolved sequence. Colours used to represent the different types of amino acids are the same as in Fig. 3 of the main text. (e) Genetic algorithm progression of this coevolution run. Shaded area for the average fitness corresponds to the standard deviation across the whole population at each round. (f) Illustration of how the systematic change from I_{ref} to I_{target} is made. (g) Comparison of the final evolved sequence with maximum fitness, O_{pred}, with the initial reference sequence O_{ref}. To illustrate the sequences in (f–g), we plot the absolute value and the change in the value of $\varepsilon_{i,\mathrm{Mpipi}}$ compared to the initial reference sequence for each residue i along the sequence.

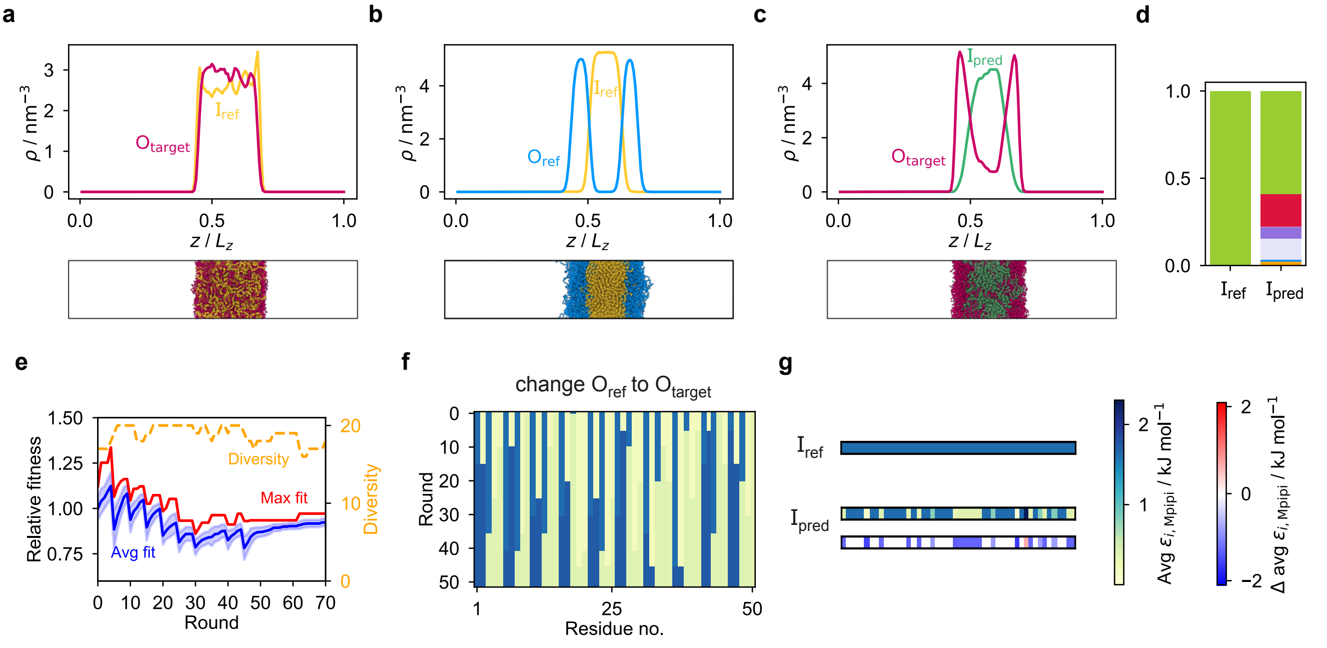

Figure S20. **Coevolution with generic sequences (II).** Analogue of Fig. S19 for a coevolution run with generic sequences where the outer sequence is systematically changed and the inner sequence is evolved. I_{ref} = F_{50}, O_{ref} = $(FAFAA)_{10}$ and O_{target} = $(YYGGR)_{10}$.

S13 FINITE-SIZE SCALING

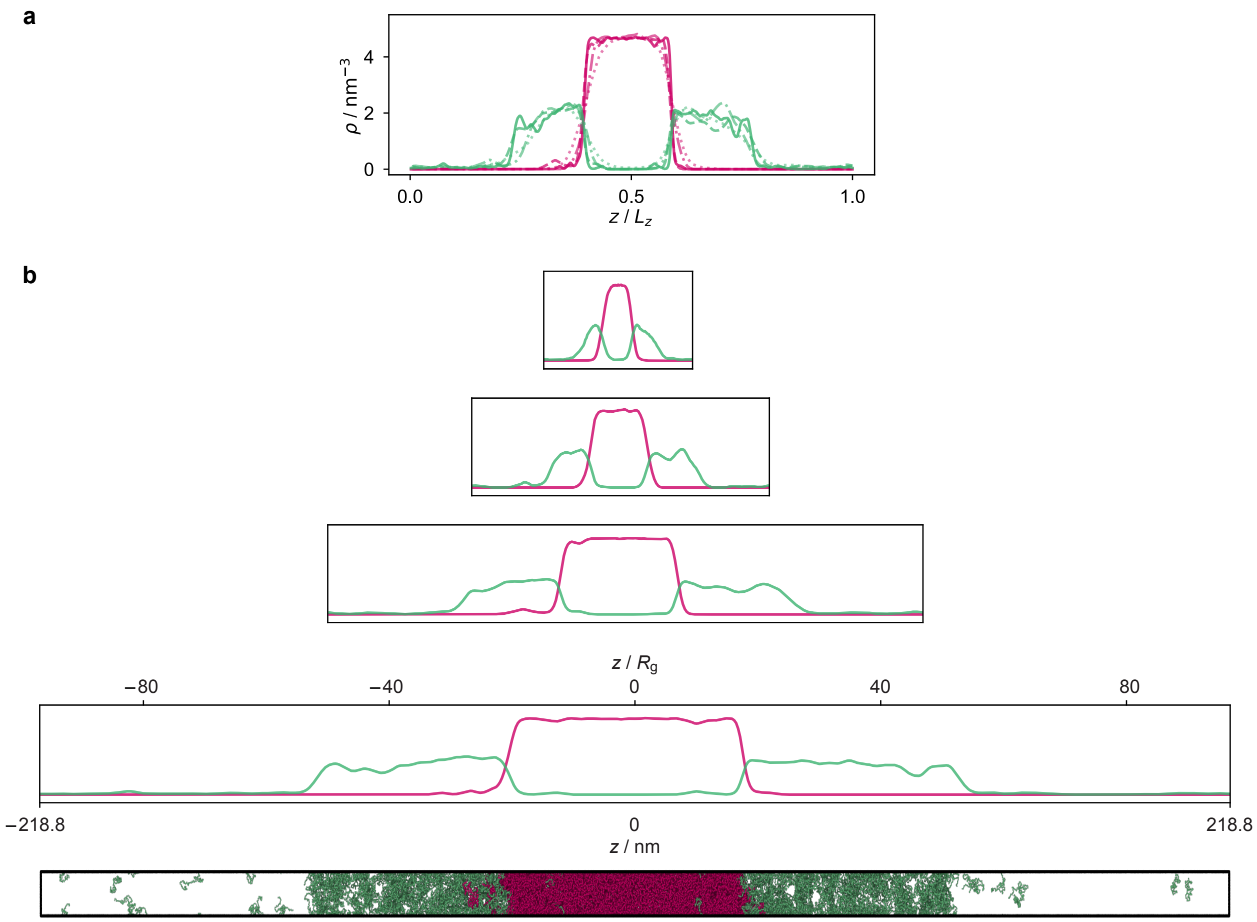

Figure S21. **Finite-size scaling (I).** Density profiles for the final system obtained in a coevolution run with A1 concentrated in the inner layer [cf. Fig. 2(b)] at two, four and eight times the size of the system reported in the main text, using the same colours and notation as in Fig. 2(b). (a) Density profiles, scaled along the horizontal axis for ease of comparison, are within typical noise across the four different system sizes. The original system size is shown in dotted lines, and systems at two, four and eight times the original size are shown in dashed-dot, dashed and solid lines, respectively. (b) Unscaled density profiles for the four system sizes. The area of the interface is kept constant at 10.9 nm × 10.9 nm, with the long axis increasing from 54.7 nm to 109.4 nm, 218.8 nm and 437.6 nm from top to bottom. The simulation snapshot included at the bottom is for the largest system size considered. For the largest system size considered, we have also shown the length in the z direction in terms of the single-molecule R_g of the outer protein at infinite dilution to illustrate the thickness of the outer phase in terms of the dimensions of a single protein. [The R_g of a protein chain of course depends on the environment that the protein is in, so the infinite-dilution R_g is just a rough approximation. The single-molecule radii of gyration for all the sequences mentioned in the main text are included in the SI alongside the sequence listing.]

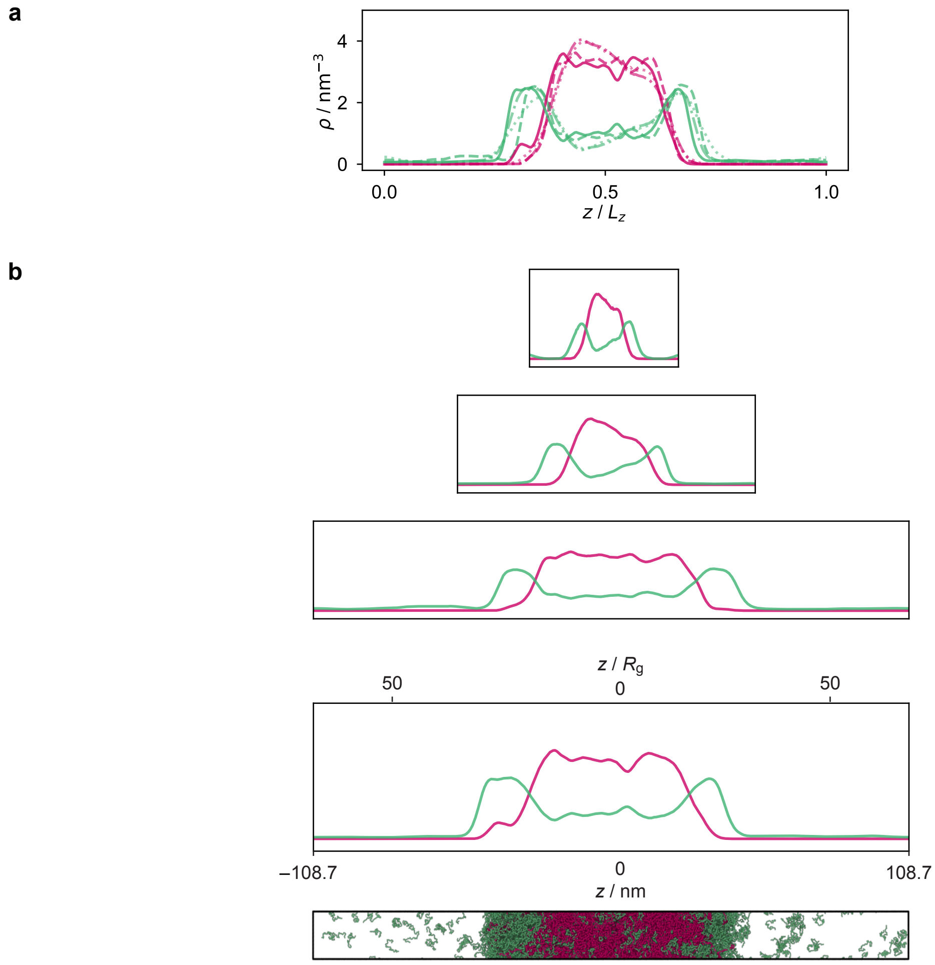

Figure S22. **Finite-size scaling (II).** Density profiles for the final system obtained in a coevolution run with A1 concentrated in the inner layer [cf. Fig. S2c] at two, four and eight times the size of the system reported in the main text, using the same colours and notation as in Fig. S2c. (a) Density profiles, scaled along the horizontal axis for ease of comparison, are within typical noise across the four different system sizes. The original system size is shown in dotted lines, and systems at two, four and eight times the original size are shown in dashed-dot, dashed and solid lines, respectively. (b) Unscaled density profiles for the four system sizes. For the first three system sizes, the area of the interface is kept constant at 10.9 nm × 10.9 nm, with the long axis increasing from 54.7 nm to 109.4 nm and 218.8 nm from top to bottom. For the largest system size, to reduce the noise in the density of the inner phase (which needs to be determined more precisely for the computation of Fig. S23(c)), the area of the interface was increased to 15.5 nm × 15.5 nm with the long axis at 217.5 nm to maintain the same overall density in the simulation box. The simulation snapshot included at the bottom is for the largest system size considered. For the largest system size considered, we have also shown the length in the z direction in terms of the single-molecule R_g of the outer protein at infinite dilution to illustrate the thickness of the outer phase in terms of the dimensions of a single protein. [The R_g of a protein chain of course depends on the environment that the protein is in, so the infinite-dilution R_g is just a rough approximation. The single-molecule radii of gyration for all the sequences mentioned in the main text are included in the SI alongside the sequence listing.]

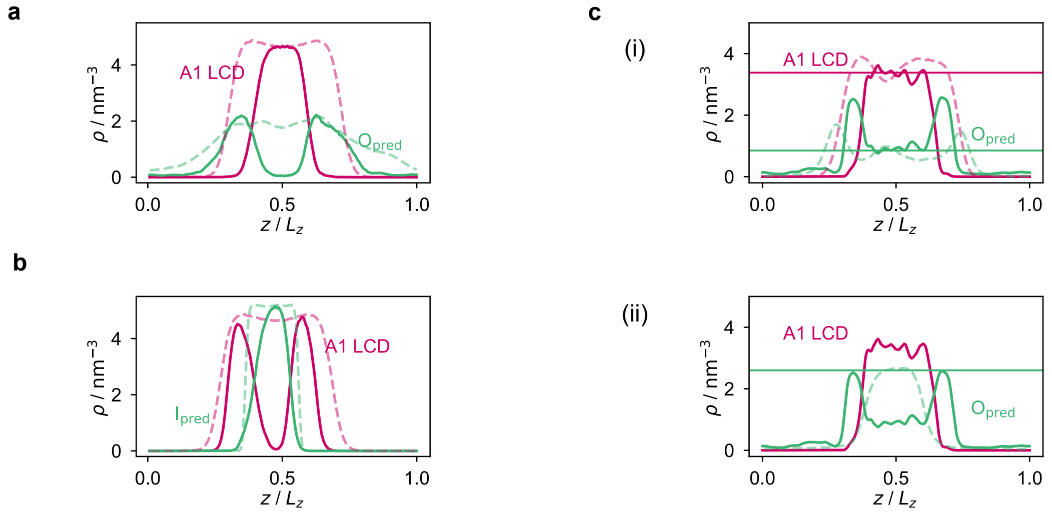

Figure S23. **Simulating individual condensed phases separately.** We have run simulations of the two condensed phases in three of our multiphasic systems [(a) = Fig. 2(b), (b) = Fig. 2(c) and (c) = Fig. S2c] separately in coexistence with the dilute phase. In (a) and (b), where the degree of multiphasicity is high, the coexisting phases are close to being pure phases. The dashed lines correspond to the density profiles of the pure components equilibrated at the same temperature as the complete multiphasic system shown in solid lines. In (c), where the degree of multiphasicity is lower, the inner phase contains both proteins in mixture (but in different proportions compared to the overall mixture), while the outer phase appears to be made up of mostly O_{pred}, and there is a non-zero density of O_{pred} in the vapour phase. We simulate (i) the inner phase and (ii) the outer phase individually in separate simulations, each in coexistence with the vapour phase. In (i) and (ii), the dashed lines correspond to the density profiles of the individual phases and the horizontal lines are drawn as a guide to indicate which densities we might expect to be the same. In all three cases, the pure-phase coexistence densities are consistent with the overall multiphasic systems.

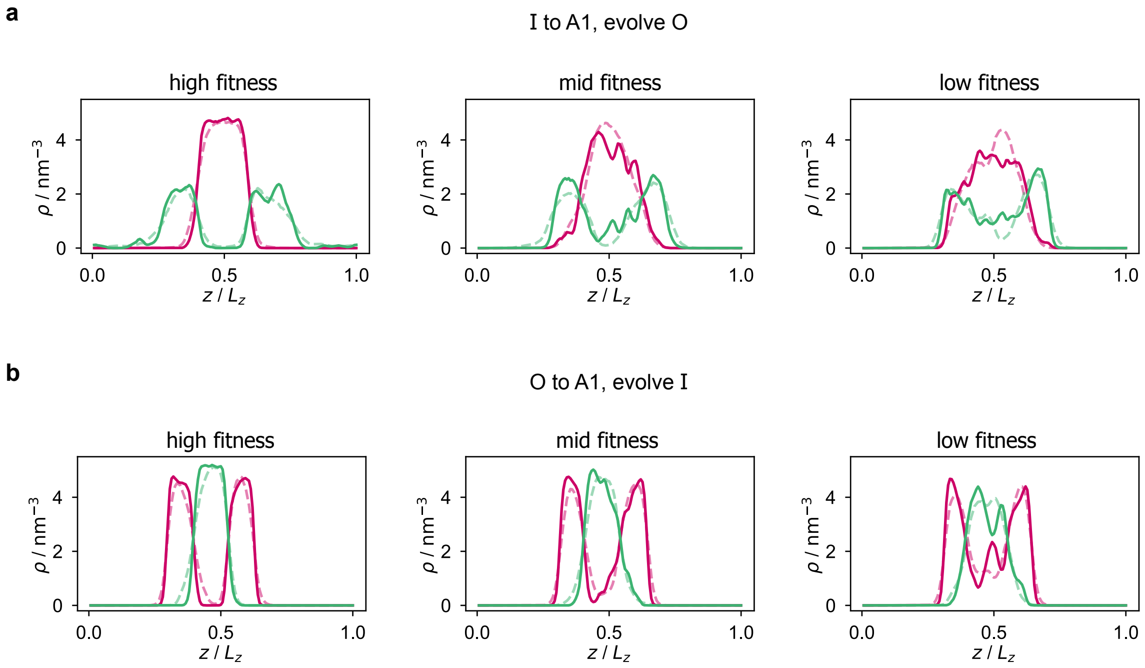

Figure S24. **Fitness function in finite-size scaling.** We have computed density profiles for systems obtained in coevolution runs with A1 [cf. Fig. 2] at twice the size of the systems reported in the main text, i.e. with 90 molecules of A1 LCD, 90 chains of 100 residues each of the partner protein in a box of size 10.9 nm × 10.9 nm × 109.4 nm. The resulting density profiles, using the same colours and notation as in [Fig. 2], are within typical noise of the analogues for the original system size (shown with dashed lines, which are scaled by a factor of two along the horizontal direction to ease comparison). The behaviour observed is similar irrespective of how fit the systems are, and the fitness ordering is maintained when the system size is doubled, suggesting that finite-size effects are unlikely to dominate the phase behaviour.

S14 LISTINGS OF PROTEIN SEQUENCES AND RADII OF GYRATION

We list below the sequences of the proteins from the figures reported of the main text. For the final evolved proteins, we highlight changes from the initial sequence in red. The sequences given here correspond to those with the maximum fitness only. In the supporting data, we provide listings of all sequences in the population of the final round of the genetic-algorithm runs.

For each sequence, we also report the radius of gyration for a single chain of the protein simulated at 250 K. The standard deviation of the radius of gyration of each protein is no larger than 0.06 nm.

S14.0.1 Figure 1

The protein O_i is $(FAFAA)_5$. The protein I_i is F_{50} with added random mutations with a probability of 0.6.

O_i	FAFAA FAFAA FAFAA FAFAA FAFAA FAFAA FAFAA FAFAA FAFAA FAFAA	R_g = 1.3 nm
$O_{f,1}$	EAAIA FAFEA NAFKA HAPAA KRAAA FAFMA YNQAA ADFAM FGHEA AEKAA	R_g = 1.6 nm
I_i	WYNMS RIFRC FFLHF FFCLL RFDFG SCFIF LFLET ENIFF FMYDF VFFFF	R_g = 1.1 nm
$I_{f,1}$	WYSMG RFFRF FFYGF FFWPG RFYFC QCFAF WFLWW EYIFF DMWDF VFFFF	R_g = 1.1 nm

S14.0.2 Figure 2

We list below the sequences of the proteins shown in Fig. 2 of the main text.

We first list the sequence of A1 LCD and its variants, using the nomenclature of Bremer and co-workers.[7] The amino-acid residues different from the wild type are highlighted in blue.

A1 LCD	MASAS SSQRG RSGSG NFGGG RGGGF GGNDN FGRGG NFSGR GGFGG SRGGG GYGGS GDGYN GFGND GSNFG GGGSY NDFGN YNNQS SNFGP MKGGN FGGRS SGPYG GGGQY FAKPR NQGGY GGSSS SSSYG SGRRF	R_g = 1.8 nm
−3R+3K	MASAS SSQRG KSGSG NFGGG RGGGF GGNDN FGRGG NFSGR GGFGG SKGGG GYGGS GDGYN GFGND GSNFG GGGSY NDFGN YNNQS SNFGP MKGGN FGGRS SGGSG GGGQY FAKPR NQGGY GGSSS SSSYG SGRKF	R_g = 2.0 nm
−6R+6K	MASAS SSQKG KSGSG NFGGG RGGGF GGNDN FGKGG NFSGR GGFGG SKGGG GYGGS GDGYN GFGND GSNFG GGGSY NDFGN YNNQS SNFGP MKGGN FGGKS SGGSG GGGQY FAKPR NQGGY GGSSS SSSYG SGRKF	R_g = 2.1 nm
+7F−7Y	MASAS SSQRG RSGSG NFGGG RGGGF GGNDN FGRGG NFSGR GGFGG SRGGG GFGGS GDGFN GFGND GSNFG GGGSF NDFGN FNNQS SNFGP MKGGN FGGRS SGGSG GGGQF FAKPR NQGGF GGSSS SSSFG SGRRF	R_g = 1.9 nm
+7K+12D	MASAD SSQRD RDDKG NFGDG RGGGF GGNDN FGRGG NFSDR GGFGG SRGDG KYGGD GDKYN GFGND GKNFG GGGSY NDFGN YNNQS SNFDP MKGGN FKDRS SGPYD KGGQY FAKPR NQGGY GGSSS SKSYG SDRRF	R_g = 1.9 nm
+7R+12D	MASAD SSQRD RDDRG NFGDG RGGGF GGNDN FGRGG NFSDR GGFGG SRGDG RYGGD GDRYN GFGND GRNFG GGGSY NDFGN YNNQS SNFDP MKGGN FRDRS SGPYD RGGQY FAKPR NQGGY GGSSS SRSYG SDRRF	R_g = 1.7 nm
−12F+12Y	MASAS SSQRG RSGSG NYGGG RGGGY GGNDN YGRGG NYSGR GGYGG SRGGG GYGGS GDGYN GYGND GSNYG GGGSY NDYGN YNNQS SNYGP MKGGN YGGRS SGGSG GGGQY YAKPR NQGGY GGSSS SSSYG SGRRY	R_g = 1.8 nm

Evolution of the outer protein when A1 LCD is at the centre. In this case, I = F_{135} and O = $(FAFAA)_{20}$.

O_i	FAFAA FAFAA FAFAA FAFAA FAFAA FAFAA FAFAA FAFAA FAFAA FAFAA FAFAA FAFAA FAFAA FAFAA FAFAA FAFAA FAFAA FAFAA FAFAA FAFAA	R_g = 1.6 nm
O_{pred} for WT	KAFMI QAFWA HPKAV MAFAA VVCHA PAYLM NLMVN FASVA LKAQW GIRAA LMTLA AAAAA FNVKA HPAAE HSHAP CAPSA FSFFA VQDAA NELAA KAFEA	R_g = 2.3 nm
O_{pred} for −3R+3K	YAHAK VLVLA MAEYK FEFAH PAAAA DVHIV TVFAA TKQAC FALGM FAPAQ QATAS HATAN FKNAA FAFAA IAGAI FASAV QPFAI AAFAM LGRAG FAHSA	R_g = 2.4 nm
O_{pred} for −6R+6K	SKTNA QAWLN FGFKG FACAA KAFTA IDHVA CIIAA EANAA VADAA APVAI TAGAA QKQMP TQNAP LKFPW FAMAR FAFAI QMVGA FYWAS DAFTS GAKAG	R_g = 2.2 nm
O_{pred} for +7F−7Y	VSLNA RAEFA NAMAA FLKKA SAFAA FAHAL AANAA FAYAL AIMQS IAQSI YYIVA VAPAR FAHAV SALSA KHFAA VATCA DAIAC FDVVA LATVA FNFAQ	R_g = 2.4 nm
O_{pred} for +7K+12D	HNHAK FAKAA HAFAA EQAPA NAYAA CAFKA NMQCA TAMAK IAWAA MAMAL DCHAA AVYDA FMPAH CAEDA GSGAA KANCA QKELA TAFSQ FFCCA FANAL	R_g = 2.4 nm
O_{pred} for +7R+12D	TDFAK TEAAI TCFEQ IASVT PLANL IDFKC RLFTR HSIIA VFFKA DAIVA LITFA FAGAI FEKSA KEVDI LHFAK IAFKA FEFDA PRKIA DAHAK TVFAA	R_g = 2.4 nm
O_{pred} for −12F+12Y	FALAG FANAV MAFFV TAYAV QANAV MASAA GAEAK FHTAA KQFMK TAFCA ISCHA KPFAA TAGAE RGFLN EVRAV GGFNV GAVAI SVHAQ FVTMA FAFMA	R_g = 2.4 nm

Evolution of the inner protein when A1 LCD is on the outside. In this case, I = $(FAFAA)_{20}$ and O = $(FIQII)_{27}$.

I_i	FAFAA FAFAA FAFAA FAFAA FAFAA FAFAA FAFAA FAFAA FAFAA FAFAA FAFAA FAFAA FAFAA FAFAA FAFAA FAFAA FAFAA FAFAA FAFAA FAFAA	R_g = 1.6 nm
I_{pred} for WT	FYMIY FAFAH RHYIW FADAA WAEQS WRFDA RLEAR HPSAR FGFAR FAWAA FWAFR FARAR KAITA WFPQA WCCYM FEFAA LAFAA WHVAA RRWAA FPQAR	R_g = 1.4 nm

S14.0.3 *Figure 3*

Evolution with partner protein fixed (Fig. 3a,b). Here, the protein O_i is $(FAFAA)_5$. The protein I_i is F_{50} with added random mutations with a probability of 0.6.

O_i	FAFAA FAFAA FAFAA FAFAA FAFAA FAFAA FAFAA FAFAA FAFAA FAFAA	R_g = 1.3 nm
$O_{f,1}$	EAAIA FAFEA NAFKA HAPAA KRAAA FAFMA YNQAA ADFAM FGHEA AEKAA	R_g = 1.6 nm
$O_{f,2}$	FAEAE FAFAA QQVAK QEKAA FAFAI MNFAA FAIAA MGNIQ HEYAA AAFAA	R_g = 1.7 nm
$O_{f,3}$	HHFAA IAVAA FAFAC KAFNP FADAA FATAQ LAYAA SAVAP FAFEG GHCSA	R_g = 1.6 nm
I_i	WYNMS RIFRC FFLHF FFCLL RFDFG SCFIF LFLET ENIFF FMYDF VFFFF	R_g = 1.1 nm
$I_{f,1}$	WYSMG RFFRF FFYGF FFWPG RFYFC QCFAF WFLWW EYIFF DMWDF VFFFF	R_g = 1.1 nm
$I_{f,2}$	WWNMS RIFRQ WFYFF FFCLV RFDHY SEFIF RFLET QWWFF FMYDF TFWFF	R_g = 1.1 nm
$I_{f,3}$	WYNMH RYPRW FFLYF FFQQL RNWFG SCFYW LFVFF FEWFF YHYYF VFFFF	R_g = 1.1 nm

Coevolution with A1 LCD (Fig. 3c,d). In coevolution runs where A1 LCD ends up at the centre, we start with I = F_{135} and O = $(FAFAA)_{20}$.

O_{ref}	FAFAA FAFAA FAFAA FAFAA FAFAA FAFAA FAFAA FAFAA FAFAA FAFAA FAFAA FAFAA FAFAA FAFAA FAFAA FAFAA FAFAA FAFAA FAFAA FAFAA	R_g = 1.6 nm
$O_{pred,1}$	KAFMI QAFWA HPKAV MAFAA VVCHA PAYLM NLMVN FASVA LKAQW GIRAA LMTLA AAAAA FNVKA HPAAE HSHAP CAPSA FSFFA VQDAA NELAA KAFEA	R_g = 2.3 nm
$O_{pred,2}$	SLIEM FAQAK EATDD MAFQL QAKQA PALKH GCNPD GQFHA FNLMR MFGAA PAMNT GATAA FALKA FTMAM GAQHH GAFNA CAMAL FTFAA FATIA SPTAS	R_g = 2.4 nm
$O_{pred,3}$	KCTAH IAFTA FTEAA NIRGI LAFLG PADAT FSFAP FAKAA EANAA MCYLK IAQKA TPVSA FAFHV VAIRC EFFKA FAALA FAKAA TTACN DSKVA FPFAA	R_g = 2.4 nm

In coevolution runs where A1 LCD ends up on the outside, we start with I = $(FAFAA)_{20}$ and O = $(FIQII)_{27}$.

I_{ref}	FAFAA FAFAA FAFAA FAFAA FAFAA FAFAA FAFAA FAFAA FAFAA FAFAA FAFAA FAFAA FAFAA FAFAA FAFAA FAFAA FAFAA FAFAA FAFAA FAFAA	R_g = 1.6 nm
$I_{pred,1}$	GNFAA WWFPY GGFAA FRYFS FWHAG FAWYA FMFAA YSFHA FSRAA FAFYG WNFRQ FEFAA FAFAC FAPAA FAFYH YARGA FAPAE NEFMA FPFYR RAFYQ	R_g = 1.3 nm
$I_{pred,2}$	FANLA EQFRW NAVAA DAFWW QYFAR NANAG FEFRL HSFYW YYNHA FHFEY FYFRR PHFDN YFFAD FAFAA FARAF PAFFR FEFRV TKFCA FLFYA FDNWW	R_g = 1.3 nm
$I_{pred,3}$	FYMIY FAFAH RHYIW FADAA WAEQS WRFDA RLEAR HPSAR FGFAR FAWAA FWAFR FARAR KAITA WFPQA WCCYM FEFAA LAFAA WHVAA RRWAA FPQAR	R_g = 1.4 nm

S14.0.4 *Figure 5*

For the case where spacers are shuffled, the amino-acid residues different from the reference evolved protein are highlighted in green.

$I_{pred,3}$	FYMIY FAFAH RHYIW FADAA WAEQS WRFDA RLEAR HPSAR FGFAR FAWAA FWAFR FARAR KAITA WFPQA WCCYM FEFAA LAFAA WHVAA RRWAA FPQAR	R_g = 1.4 nm
I_{sorted}	IIIVL LKHHH HTMMA AAAAA AAAAA AAAAA AAAAA AAAAA AAASS CCPPP DDEEE RRRRR RRRRR RRGQQ QFFFF FFFFF FFFFF FFYYY YWWWW WWWWW	R_g = 1.4 nm
$O_{pred,1}$	KAFMI QAFWA HPKAV MAFAA VVCHA PAYLM NLMVN FASVA LKAQW GIRAA LMTLA AAAAA FNVKA HPAAE HSHAP CAPSA FSFFA VQDAA NELAA KAFEA	R_g = 2.3 nm
$O_{shuffled\ spacers}$	ASFAV QKFWL MPECL HAFPA SKLAA DAYAM NGVAN FAMVM APSQW EARAM HAAAA AILLT FNAVA APVPA CAAAA KKSAL FEFFA HQAAA NAVIV KAFHH	R_g = 2.3 nm

REFERENCES

[1]Y.-H. Lin, J. P. Brady, J. D. Forman-Kay, and H. S. Chan, "Charge pattern matching as a 'fuzzy' mode of molecular recognition for the functional phase separations of intrinsically disordered proteins," New J. Phys. **19**, 115003 (2017).

[2]T. Pal, J. Wessén, S. Das, and H. S. Chan, "Subcompartmentalization of polyampholyte species in organelle-like condensates is promoted by charge-pattern mismatch and strong excluded-volume interaction," Phys. Rev. E **103**, 042406 (2021).

[3]R. K. Das and R. V. Pappu, "Conformations of intrinsically disordered proteins are influenced by linear sequence distributions of oppositely charged residues," Proc. Natl Acad. Sci. U.S.A. **110**, 13392–13397 (2013).

[4]L. Sawle and K. Ghosh, "A theoretical method to compute sequence dependent configurational properties in charged polymers and proteins," J. Chem. Phys. **143**, 085101 (2015).

[5]E. W. Martin, A. S. Holehouse, I. Peran, M. Farag, J. J. Incicco, A. Bremer, C. R. Grace, A. Soranno, R. V. Pappu, and T. Mittag, "Valence and patterning of aromatic residues determine the phase behavior of prion-like domains," Science **367**, 694–699 (2020).

[6]S. M. Lichtinger, A. Garaizar, R. Collepardo-Guevara, and A. Reinhardt, "Targeted modulation of protein liquid–liquid phase separation by evolution of amino-acid sequence," PLOS Comput. Biol. **17**, e1009328 (2021).

[7]A. Bremer, M. Farag, W. M. Borcherds, I. Peran, E. W. Martin, R. V. Pappu, and T. Mittag, "Deciphering how naturally occurring sequence features impact the phase behaviours of disordered prion-like domains," Nat. Chem. **14**, 196–207 (2021).
